# Nanoscale Metal‐Organic Framework‐Based Self‐Monitoring Oxygen Economizer and ROS Amplifier for Enhanced Radiotherapy‐Radiodynamic Therapy

**DOI:** 10.1002/advs.202503582

**Published:** 2025-06-23

**Authors:** Shiye Du, Qiang Wen, Ting Han, Jiongyu Ren, Mingyu Wang, Yunpeng Dai, Xiaoguang Ge, Lu Li, Junzhi Liu, Shi Gao

**Affiliations:** ^1^ Department of Nuclear Medicine China‐Japan Union Hospital of Jilin University Changchun 130033 China; ^2^ Department of Radiotherapy ‐1 and Department of Radiotherapy ‐4 Jilin Province Cancer Hospital Changchun 130000 China

**Keywords:** hypoxia reversion, metal‐organic framework, radiodynamic therapy, radiotherapy, reactive oxygen species

## Abstract

Radiotherapy‐radiodynamic therapy (RT‐RDT) has emerged as a promising approach due to its remarkable anticancer efficacy. However, hypoxic tumor microenvironment and insufficient energy deposition severely reduce the radiotherapy outcomes. Herein, lonidamine (LND)‐loaded Fe(III) metal‐Pt(II) porphyrin framework (FPTM‐LP) is designed for radiosensitization through hypoxia alleviation and reactive oxygen species (ROS) amplification. Specifically, the high‐Z element Pt effectively absorbs X‐ray photons to generate •OH for RT while transferring X‐ray energy to porphyrin (TCPP), which stimulates the generation of ^1^O_2_ for RDT. Moreover, by the inhibition of oxidative phosphorylation, the weak acidity and glutathione (GSH)‐responsive release of LND achieves O_2_ economization, which promotes the generation of ROS and stabilizes DNA damage. Intriguingly, Pt(II)TCPP exhibits the capability of O_2_ concentration‐dependent near infrared luminescence imaging‐guided RT‐RDT. Moreover, the decomposition of FPTM‐LP under high concentration of GSH generates large amounts of Fe^2+^, leading to the augmented production of •OH via Fenton reactions. In brief, this work presents a radiosensitizer with a unique and efficient mechanism of self‐monitoring hypoxia alleviation and increasing ROS levels for enhanced cancer RT‐RDT. The radiosensitization potency of FPTM‐LP is confirmed in tumor‐bearing mice in vivo, proclaiming a new strategy for enhancing therapeutic efficacy and minimizing side effects.

## Graphical abstract

A Fe (III)–Pt (II) porphyrin framework‐based radiosensitizer (FPTM‐LP) is constructed to serve as an oxygen economizer, hypoxia self‐monitoring device, and reactive oxygen species (ROS) amplifier. The glutathione (GSH)/pH‐responsive release of lonidamine achieves oxygen‐economization to combat hypoxia‐induced radioresistance guided by near infrared luminescence imaging. Pt (II) porphyrin and Fe^2+^ synergistically amplify the ROS generation to enhance radiotherapy‐radiodynamic therapy.

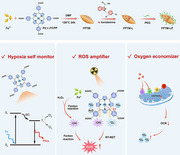

## 1 Introduction

Radiotherapy (RT) is a first‐line clinical therapy for cancer that directly causes DNA damage through high‐energy ionizing radiation or indirectly leads to cell death by generating reactive oxygen species (ROS).^[^ ^1^ ^]^ High ROS levels play an indispensable role in RT. However, inadequate ROS generation due to insufficient energy deposition and hypoxia‐induced radioresistance severely hinders the efficacy of RT.^[^ ^2^ ^]^ Consequently, a fundamental strategy to enhance the efficacy of RT is to increase the generation and effectiveness of ROS through various methods.^[^ ^3^ ^]^

In recent decades, the development of radiosensitizers has focused on high‐Z metal elements‐based nanomaterials, including hafnium (Hf),^[^ ^4^ ^]^ gold (Au),^[^ ^5^ ^]^ neodymium (Nd), and gadolinium (Gd).^[^ ^6^ ^]^ Leveraging their exceptional photoelectric and Compton effects, high‐Z elements possess enhanced capabilities to absorb X‐ray energy, thereby promoting therapeutic outcome of RT.^[^ ^7^ ^]^ Among the various nanomaterials used for radiosensitization, metal‐organic frameworks (MOFs) containing high‐Z elements are recognized as a class of inorganic‐organic hybrid nanomaterials that exhibit excellent radiosensitization effects due to their substantial dose enhancement effect and superior mass attenuation coefficients.^[^ ^8^ ^]^ Recent studies have reported that high‐Z metal‐porphyrin frameworks can be utilized as X‐ray scintillators to achieve a unique RT‐radiodynamic therapy (RDT) process.^[^ ^9^ ^]^ High‐Z elements efficiently absorb X‐ray energy, augmenting water radiolysis to increase the production of hydroxyl radicals (•OH) for RT, while facilitating energy transfer to photosensitive linkers to generate singlet oxygen (^1^O_2_) for RDT.^[^ ^10^ ^]^ This synergistic effect of RT‐RDT significantly amplifies X‐ray radiation‐induced damage to tumors.

Although radiation enhancers can improve RT‐RDT efficacy, tumor hypoxia remains a key factor affecting RT‐RDT outcomes. The hypoxic tumor microenvironment (TME) is detrimental to ROS generation and can nullify the “ radiation damage fixation ” effect of oxygen (O_2_) during RT‐RDT.^[^ ^11^ ^]^ Additionally, reduced O_2_ levels in tumor leads to the upregulated expression of hypoxia‐inducible factor 1‐alpha (HIF‐1α), which facilitates tumor migration, invasion, recurrence, and immune evasion.^[^ ^12^ ^]^ To date, various strategies focusing on endogenous O_2_ generation and exogenous O_2_ perfusion have been developed to reverse tumor hypoxia, such as metal‐based nanozymes and perfluorocarbons.^[^ ^13^ ^]^ However, these strategies are not highly efficient because only a limited amount of O_2_ can be generated or delivered.^[^ ^14^ ^]^ Recently, an alternative approach has been proposed to inhibit mitochondrial‐associated oxidative phosphorylation (OXPHOS) and reduce the oxygen consumption rate (OCR), thereby conserving more O_2_ to overcome tumor hypoxia.^[^ ^15^ ^]^ Reports suggested that reducing O_2_ consumption is more effective than increasing O_2_ supply in alleviating tumor hypoxia.^[^ ^16^ ^]^ Moreover, accurate evaluation of local O_2_ levels is beneficial for precisely predicting treatment outcomes and modifying therapeutic regimens.^[^ ^17^ ^]^ To date, a series of biological sensors have been designed for O_2_ imaging, which hold significant potential for cancer diagnosis and therapeutic efficacy evaluation.^[^ ^18^ ^]^ However, the construction of self‐monitored O_2_‐economized platforms for augmenting RT‐RDT is relatively scarce, highlighting the importance of designing nanoplatforms for O_2_‐sensing while incorporating radiosensitizing capabilities.

**Scheme 1:**
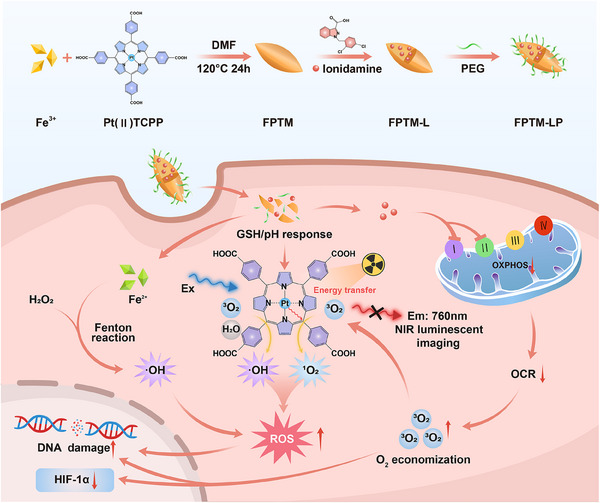
Schematic of FPTM‐LP preparation and the mechanism for hypoxia imaging and O_2_‐economized enhanced RT‐RDT against breast cancer.

Herein, we developed a nanoscale MOF (FPTM‐LP) as self‐monitored O_2_ economizer and ROS amplifier for radiosensitization (**Scheme** **1**). By coordinating Fe(III) with Pt(II) meso‐tetra (4‐carboxyphenyl) porphine (Pt(II)TCPP) and efficiently loading lonidamine (LND), FPTM‐LP modifying with polyethylene glycol (PEG) was constructed for enhanced RT‐RDT. Specifically, Pt effectively absorbs X‐ray photons to generate •OH for RT while transferring X‐ray energy to TCPP to stimulate the generation of ^1^O_2_ for RDT. Concurrently, Fe(II), which originated from the redox reaction between Fe(III) and glutathione (GSH), amplified the generation of ROS by Fenton reactions. Additionally, the weak acidity/GSH‐responsive release of LND inhibited mitochondrial complexes I and II,^[^ ^19^ ^]^ further disrupting OXPHOS and reducing endogenous O_2_ consumption, thereby achieving O_2_‐economization. Alleviating hypoxia promoted the generation and effectiveness of ROS, stabilized DNA damage, and downregulated the expression of HIF‐1α. Intriguingly, the O_2_ concentration‐dependent luminescence emission capability of Pt(II)TCPP enabled real‐time and reversible O_2_ sensing. This study presented a nanoplatform that integrates hypoxia imaging, O_2_‐economized enhanced RT‐RDT, synergistically enhancing the ROS generation and providing a novel assessment and resolution strategy for hypoxia‐induced radioresistance.

## 2 Results and Discussion

### 2.1 Synthesis and Characterization of FPTM‐LP

**Figure 1:**
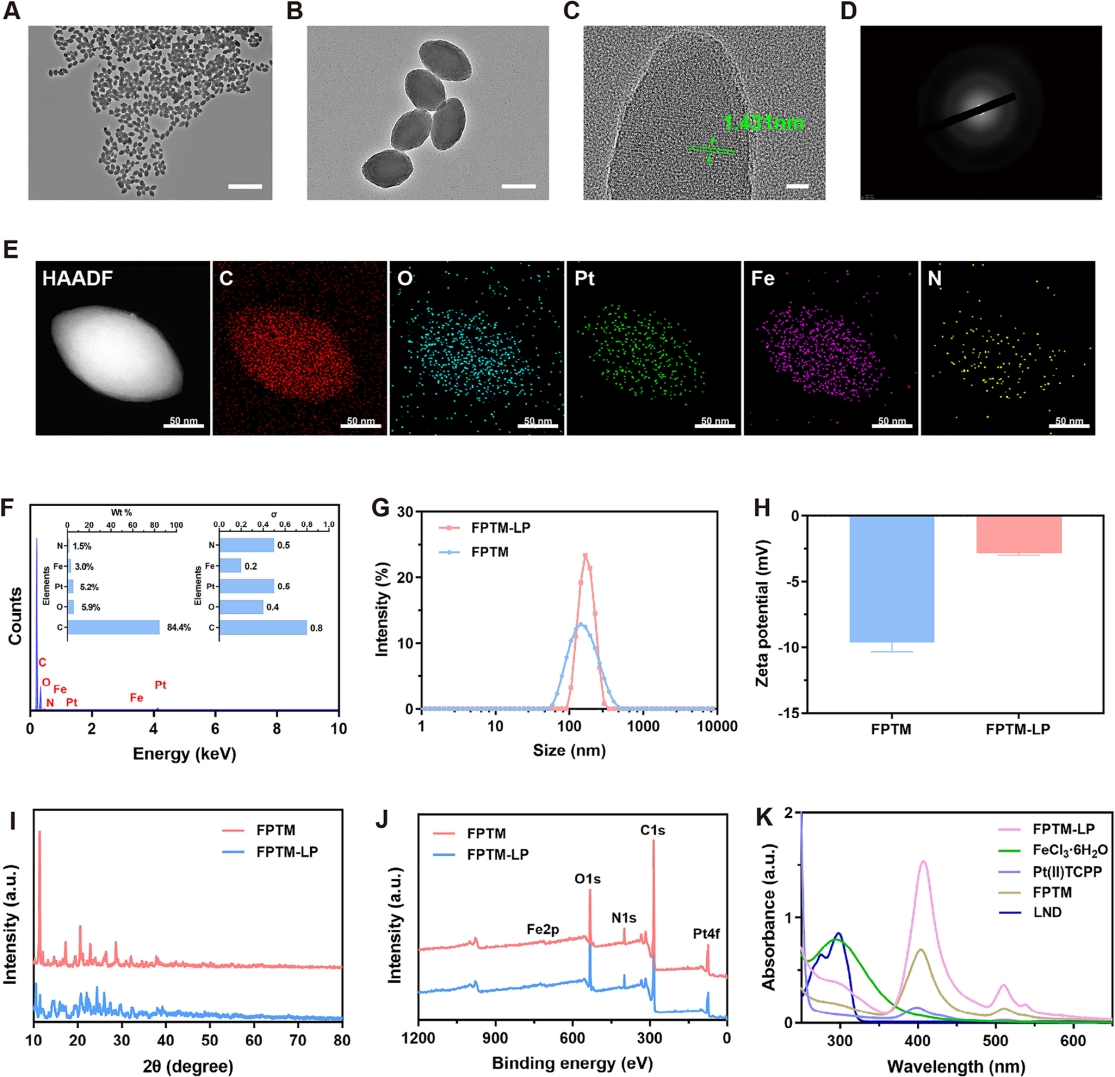
Synthesis and characterization of FPTM‐LP. A) TEM image of FPTM (Scale bar: 1.0 µm). B) TEM image of FPTM‐LP (Scale bar: 100 nm). C) HRTEM image of FPTM (Scale bar: 10 nm). D) Electron diffraction image of FPTM. E) High‐angle annular dark‐field (HAADF) TEM image of FPTM and element mapping of C, O, Pt, Fe, and N (Scale bar: 50 nm). F) Quantitative elemental analysis of FPTM through EDS. G) The hydrodynamic size distribution of FPTM and FPTM‐LP. H) Zeta potentials of FPTM and FPTM‐LP. I) XRD pattern of FPTM and FPTM‐LP. J) XPS spectra of FPTM and FPTM‐LP. K) UV–vis absorption spectra of FeCl_3_·6H_2_O, Pt(II)TCPP, LND, FPTM and FPTM‐LP.

Nanoscale FPTM was synthesized via a one‐pot solvothermal method by coordinating Fe(III) with Pt(II)TCPP ligands, using benzoic acid as a regulator. By adjusting the reaction temperature, nanoparticles with an appropriate size were prepared (Figure S1, Supporting Information). Transmission electron microscope (TEM) image revealed that FPTM presented a uniform olive‐shaped morphology (**Figure** **1**A). The crystallographic property of FPTM with a lattice plane spacing of 1.431 nm was observed by high‐resolution TEM (Figure 1C), and the selected area electron diffraction (SAED) pattern of FPTM was shown in Figure 1D. Additionally, TEM elemental mapping confirmed a uniform distribution of C, O, Pt, Fe, and N in the nanoparticles (Figure 1E), indicating the successful construction of FPTM. This finding was supported by energy dispersive X‐ray spectroscopy (EDS) (Figure 1F). By dynamic light scattering (DLS) measurement, FPTM exhibited a hydrodynamic size of 140 nm (Figure 1G). Furthermore, the loading of LND and modification with PEG did not significantly alter the olive‐shaped morphology observed by TEM (Figure 1B). However, PEG modification shifted the zeta potential from ‐9.64 to ‐2.83 mV (Figure 1H), and resulted in a slight increase in hydrodynamic size to 160 nm. X‐ray diffraction (XRD) analysis demonstrated that FPTM‐LP possessed the same characteristic peaks of crystal structure as FPTM in the range of 10° to 35° (Figure 1I). Subsequently, the chemical composition of FPTM was analyzed using X‐ray photoelectron spectroscopy (XPS). The Fe 2p characteristic peak suggested that Fe^3^⁺ was the predominant species (Figure 1J). In addition, the PEG modification caused the characteristic peak signals of FPTM‐LP to be lower than those of FPTM. The ultraviolet‐visible (UV‐vis) absorption spectra of FPTM displayed the presence of the same characteristic absorption peak as that of Pt(II)TCPP (Figure 1K), which could be attributed to the successful coordination between Fe(III) with the carboxyl groups of Pt(II)TCPP. These results proved the successful construction of FPTM‐LP. Additionally, FPTM‐LP demonstrated remarkable stability in neutral phosphate buffered saline (PBS), Dulbecco's modified Eagle's medium (DMEM) containing 10% fetal bovine serum (FBS), mouse serum and distilled water, effectively avoiding nonspecific drug release (Figure S2, Supporting Information).

### 2.2 GSH/pH‐Responsive Behavior, O_2_‐Sensing Ability, and ROS Generation of FPTM‐LP

**Figure 2:**
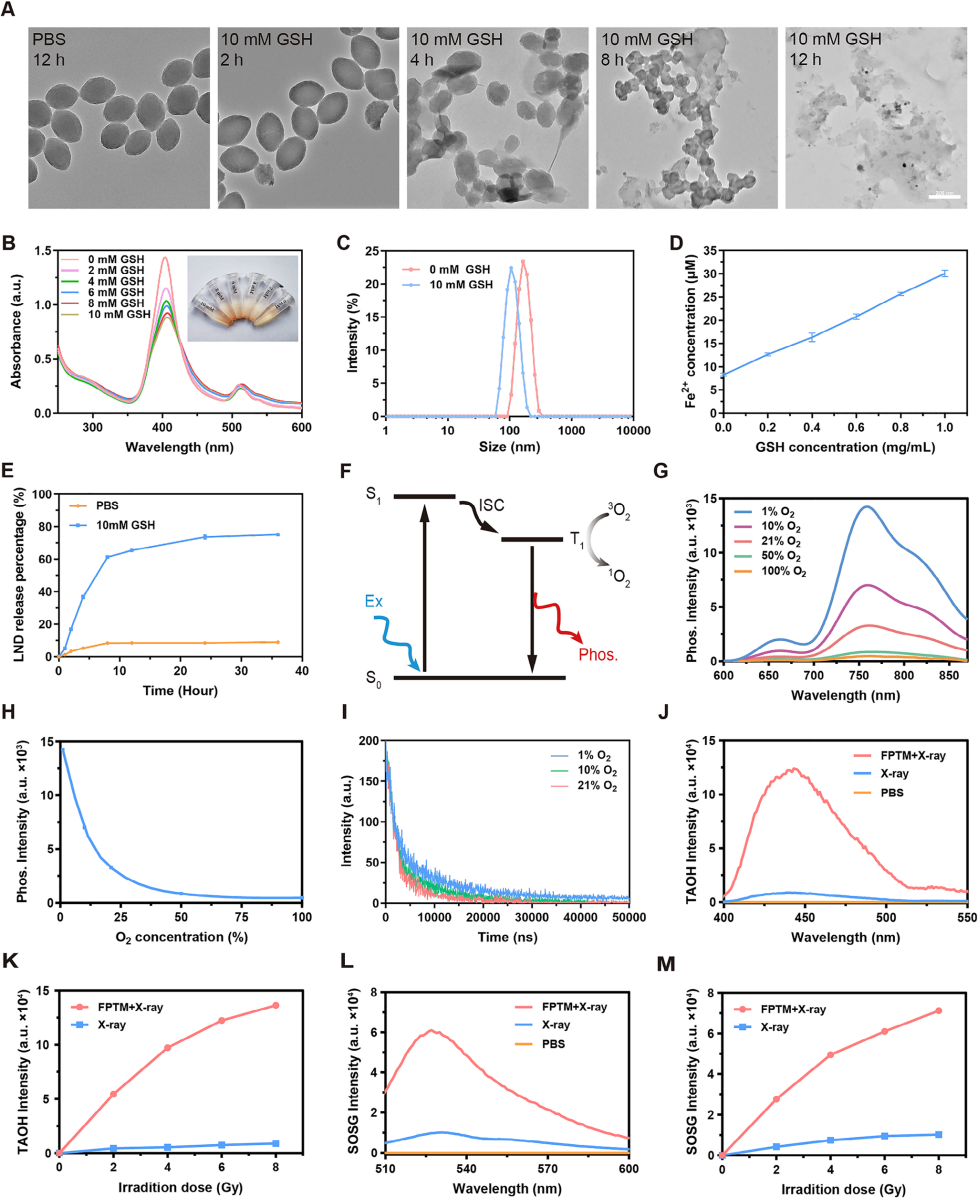
The ability of GSH response, O_2_‐sensing and radiosensitization in vitro. A) TEM images illustrating the effect of GSH on the morphology of FPTM‐LP at various exposure times (Scale bar: 200 nm). B) UV‐vis absorption spectra and images (inset) of FPTM‐LP after treatment with different concentrations of GSH. C) The hydrodynamic size changes of FPTM‐LP after treatment with GSH. D) The release of Fe^2+^ after treatment with different concentrations of GSH (*n* = 3). E) Release curves of LND after treatment with GSH (*n* = 3). F) Schematic diagram illustrating the O_2_ concentration‐dependent emission mechanism of FPTM. G) O_2_‐dependent emission spectra of FPTM‐LP. H) Changes in luminescence intensity at different O_2_ concentrations (*n* = 3). I) Changes in phosphorescence lifetime at different O_2_ concentrations. J) Fluorescence spectra of TAOH under X‐ray irradiation in the presence of FPTM, demonstrating the generation of •OH. K) The·•OH generation ability of FPTM under different irradiation doses. L) Fluorescence spectra of SOSG under X‐ray irradiation in the presence of FPTM, demonstrating the generation of ^1^O_2_. M) The ^1^O_2_ generation ability of FPTM under different irradiation doses. Data were expressed as mean ± SD.

It is well‐known that the TME‐responsive drug release is crucial for minimizing serious side effects and achieving precise cancer treatment,^[^ ^20^ ^]^ thus the GSH/pH‐responsive behavior of FPTM‐LP was explored. Further insights were obtained through TEM, which revealed the morphological change of FPTM‐LP at different time intervals (**Figure** **2**A). In neutral PBS solution, FPTM‐LP maintained its olive‐shaped morphology after 12 h. However, after incubation with GSH solution (10 mm) for 2 h, a degree of shell degradation was observed. Over time, the olive‐shaped nanoparticles gradually disintegrated and transformed into irregular fragments. As shown in Figure 2B, GSH induced significant precipitation in the FPTM‐LP solution, accompanied by a concentration‐dependent decrease of UV–vis absorption signal intensity. DLS measurements showed that the hydrodynamic size of FPTM‐LP treated with GSH decreased significantly, implying the potential of controlled drug release (Figure 2C). The redox interaction between Fe^3+^ and GSH also resulted in the generation of Fe^2+^, which could trigger the Fenton reaction to elevate the ROS levels in the presence of H_2_O_2_. Notably, as illustrated in Figure 2D, the concentration of released Fe^2+^ was directly correlated with the concentration of GSH. We also found that an acidic environment could induce the degradation of FPTM‐LP (Figure S3A, Supporting Information). More importantly, the presence of GSH in the acidic environment accelerated the disintegration of FPTM‐LP (Figure S3B, Supporting Information). Additionally, we also examined the GSH/pH‐responsive release of LND from FPTM‐LP. As displayed in Figure 2E, approximately 75% of LND was released after 36 h under high GSH levels, whereas only about 9% was released under physiological conditions. Moreover, the release of LND was accelerated in an acidic environment with high GSH levels (Figure S4, Supporting Information). Collectively, these findings confirmed the robust GSH/pH response of FPTM‐LP, which facilitated the controlled release of LND and Fe^2^⁺.

Since hypoxia is a major contributor of radioresistance, pre‐assessing the degree of hypoxia within tumors is crucial for predicting the RT efficacy and formulating effective treatment planning.^[^ ^21^ ^]^ It is widely recognized that the ground‐state electrons (S_0_) of metalloporphyrin can be excited to the lowest singlet excited state and promptly transition to the triplet state via intersystem crossing (Figure 2F).^[^ ^22^ ^]^ Upon contact with O_2_ molecules, some excited metalloporphyrin undergoes quenching through collision with ^3^O_2_, generating active ^1^O_2_ and leading to low emission intensity of the triplet state. Consequently, as the O_2_ concentration increases, the luminescence yield decreases.^[^ ^23^ ^]^ This O_2_‐induced quenching effect can be harnessed for direct and reversible measurements of O₂ levels.^[^ ^24^ ^]^ It is believed that Pt(II)TCPP‐based MOF possessed O_2_‐sensing capability. As shown in Figure 2G, a luminescence emission peak was detected. Along with the O_2_ concentration gradually increased from 1% to 100%, the emission intensity at 760 nm dropped sharply, with the intensity in 1% O_2_ being nearly 30 times higher than that in 100% O_2_ (Figure 2H). Furthermore, the phosphorescence lifetime decreased from 0.67 µs in 1% O_2_ to 0.48 µs in 21% O_2_ (Figure 2I). These data implied that FPTM was suitable for cellular hypoxia imaging and held potential for the development of a theranostic platform.

Given the presence of high‐Z element (Pt) and photosensitizng linker (TCPP), we proposed that FPTM served as a potent trigger for RT‐RDT. To investigate the ROS generation ability of FPTM under X‐ray irradiation, disodium terephthalate (TA) was chosen to assess the generation of •OH. Notably elevated fluorescence signals of oxidated TA (TAOH) were observed in the FPTM group compared to the PBS group (Figure 2J), suggesting a significant enhancement in the generation of •OH under X‐ray irradiation. A positive linear relationship between the amount of •OH generation with the radiation dose was induced by FPTM, while the change in the control group was minimal (Figure 2K). In addition, the capability of FPTM for boosting ROS generation was also studied by mixing H_2_O_2_, GSH, and FPTM. The coexistence of these three substances served as a prerequisite for detecting a robust fluorescence signal, which suggested that Fe^2^⁺, released from FPTM via the reduction by GSH, underwent a Fenton reaction with H_2_O_2_ to produce •OH (Figure S5, Supporting Information). Additionally, to explore the radiodynamic properties of FPTM, singlet oxygen sensor green (SOSG) was utilized to detect the ¹O_2_ generation. As expected, X‐ray irradiation caused a dramatic rise in fluorescence signal intensity in the FPTM group compared to negligible ¹O_2_ production in the PBS group (Figure 2L). Moreover, the production of ¹O_2_ increased with increasing X‐ray doses (Figure 2M). Electron spin resonance (ESR) was further employed to evaluate the generation of ¹O_2_ by FPTM under X‐ray irradiation. Consistent with the aforementioned results, a characteristic 1:1:1 peak of ^1^O_2_ was detected in the FPTM + X‐ray group, whereas no such peaks were observed in the PBS and X‐ray groups (Figure S6, Supporting Information). The typical 1:2:2:1 peak corresponding to the •OH was detected in both the X‐ray and FPTM + X‐ray groups. Notably, the peak intensity in the FPTM + X‐ray group was significantly higher than that in the X‐ray group (Figure S7, Supporting Information). Overall, Pt efficiently absorbed radiation energy, enhancing water radiolysis to generate •OH for improved RT outcomes. Meanwhile, Pt transferred radiation energy to the porphyrin‐based photosensitizer, facilitating the production of ¹O_2_ for RDT.

### 2.3 In Vitro Radiosensitizing Mechanisms of FPTM‐LP

**Figure 3:**
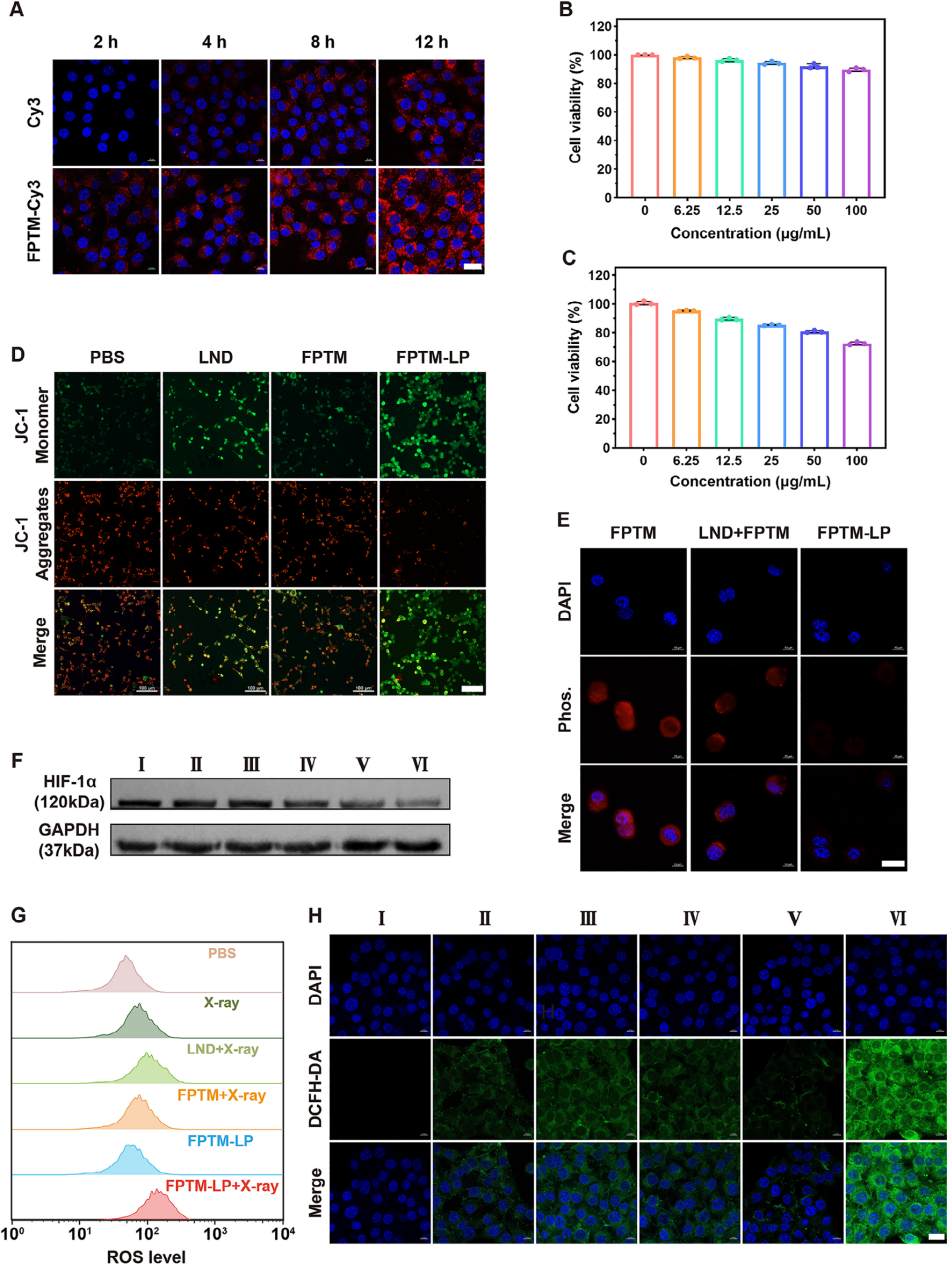
Cellular uptake, biocompatibility, hypoxia relief, and ROS generation capability of FPTM‐LP. A) CLSM images of 4T1 cells incubated with FPTM‐Cy3, illustrating the cellular uptake at different time intervals (Scale bar: 20 µm). B) Cell viability of L929 cells after treatment with different concentrations of FPTM‐LP, indicated that FPTM‐LP exhibits favorable biocompatibility with L929 cells (*n* = 3). C) Cell viability of 4T1 cells after treatment with different concentrations of FPTM‐LP, indicated that FPTM‐LP exhibits specific cytotoxicity to 4T1 cells (*n* = 3). D) CLSM images illustrating the MMP changes under different treatments (Scale bar: 100 µm). E) CLSM images of 4T1 cells stained by DAPI (green) and FPTM (red) under hypoxic conditions, indicating cellular O₂ levels (Scale bar: 20 µm). F) Western blot analysis of HIF‐1α expression under hypoxic conditions. G) Flow cytometry results of ROS levels under hypoxic conditions. H) CLSM images of ROS levels under hypoxic conditions detected by DCFH‐DA (I: PBS; II: X‐ray; III: LND + X‐ray; IV: FPTM + X‐ray; V: FPTM‐LP; VI: FPTM‐LP + X‐ray. Scale bar: 20 µm). Data were expressed as mean ± SD.

Before applying FPTM‐LP for in vitro antitumor effects, it was essential to explore the endocytosis characteristics of nanoparticles by cells. To this end, cyanine dye Cy3‐labeled FPTM (FPTM‐Cy3) was utilized to investigate the internalization by 4T1 cells at specific time intervals. As shown in **Figure** **3**A, mouse breast cancer cell line (4T1) cells treated with FPTM‐Cy3 exhibited stronger red fluorescence compared to those treated with free Cy3, confirming that the FPTM loading enhances cellular internalization of the therapeutic agent. Additionally, with longer incubation times, the fluorescence intensity increased, demonstrating a time dependent endocytosis manner.

The biocompatibility and specific cytotoxicity of nanoparticles are crucial for in vivo applications and clinical translation. Taking all this into consideration, the cytotoxicity of FPTM‐LP towards 4T1 cells and mouse fibroblast cell line (L929) cells was assessed through cell counting kit‐8 (CCK‐8) assays. The survival rate of L929 cells cocultured with FPTM‐LP remained above 85% even at a high concentration of 100 µg mL^−1^, proving the satisfactory biocompatibility of FPTM‐LP (Figure 3B). In contrast to the outcomes observed with L929 cells, FPTM‐LP significantly inhibited the viability of 4T1 cells at the same concentration (Figure 3C), implying its specific tumor‐killing ability. In addition, the FPTM‐LP‐induced cytotoxicity against 4T1 cells also showed a dosage‐dependent behavior. These results demonstrated that FPTM‐LP is compatible with normal cells at appropriate concentrations but toxic to 4T1 cells due to GSH/pH‐responsive ability of FPTM‐LP.

The hypoxic TME primarily arises from excessive O_2_ consumption by proliferating tumor cells through the electron transport chain and OXPHOS during mitochondrial aerobic respiration.^[^ ^25^ ^]^ Thus, inhibiting mitochondrial aerobic respiration can effectively alleviate hypoxia in TME and enhance radiosensitivity. LND inhibits mitochondrial complexes I and II, disrupting electron transfer and causing mitochondrial dysfunction, which manifests as depolarization of the mitochondrial membrane potential (MMP) as well as a reduction in intracellular adenosine triphosphate (ATP) production. 5,5′,6,6′‐tetrachloro‐1,1′,3,3′‐tetraethylbenzimidazolo‐carbocyanine iodide (JC‐1) is a common indicator of MMP.^[^ ^26^ ^]^ Specifically, at high MMP, JC‐1 forms aggregate and emits red fluorescence; while at low MMP, it exists as monomers, emitting green fluorescence. The results manifested that bright red fluorescence emission was observed in the PBS or FPTM treated cells (Figure 3D). As expected, pronounced depolarization of MMP was detected in the FPTM‐LP treated cells, suggesting intense mitochondrial dysfunction. In contrast, minimal green fluorescence signal was observed in the LND treated cells, which was attributed to the limited uptake of free LND. The impact of mitochondrial respiratory dysfunction can be further explored by detecting changes in intracellular ATP levels. As shown in Figure S8 (Supporting Information), FPTM‐LP significantly reduced the production of ATP, which was attributed to the obstruction of cellular energy metabolism induced by FPTM‐LP‐mediated mitochondrial respiratory inhibition. These results confirmed that FPTM‐LP affected mitochondrial integrity and function, potentially elevating intracellular O_2_ levels through O_2_‐economization.

To evaluate its bioimaging potential, FPTM was utilized for cellular imaging under different O_2_ concentrations. As shown in Figure S9 (Supporting Information), under normoxic conditions (21% O_2_), no discernible luminescence signal was observed from the 4T1 cells. However, the cells exhibited strong luminescence emission under hypoxic conditions and the luminescence intensity diminished as the O_2_ concentration increased. Furthermore, the self O_2_‐sensing property was leveraged to explore the impact of FPTM‐LP on intracellular O_2_ levels. Distinctly, intense red luminescence was significantly quenched in 4T1 cells after treatment with FPTM‐LP (Figure 3E), suggesting that O_2_ levels were restored to normoxia, attributed to the inhibition of intracellular O_2_ consumption. It is noteworthy that LND did alleviate cellular hypoxia somewhat, but the effect was far from sufficient. Subsequent western blot results revealed that HIF‐1α expression was downregulated after treatment with FPTM‐LP (Figure 3F and Figure S10, Supporting Information), which further verified the alleviation of hypoxia. In summary, FPTM‐LP had the potential to overcome radioresistance by disrupting mitochondrial function, modulating intracellular O₂ levels, and downregulating the HIF‐1α pathway.

RT‐RDT exerted its therapeutic effect primarily by ROS‐induced biomolecules damage. Therefore, we evaluated the intracellular ROS generation by FPTM‐LP under X‐ray irradiation, using 2′, 7′‐dichlorodihydrofluorescein diacetate (DCFH‐DA) as a fluorescence probe.^[^ ^27^ ^]^ Under hypoxic conditions, flow cytometry analysis revealed increased ROS levels across all X‐ray radiation‐exposed groups, with the highest levels observed in the FPTM‐LP + X‐ray group (Figure 3G). FPTM‐LP resulted in an approximately 1.5‐fold increase in the ROS levels compared to X‐ray monotherapy. Additionally, the combination of FPTM‐LP and X‐ray induced the strongest fluorescence emission observed by confocal laser scanning microscopy (CLSM) (Figure 3H), providing new evidence for the enhancement of ROS production by FPTM‐LP. Moreover, a slight increase of fluorescence signal was discovered in the FPTM‐LP without X‐ray radiation group due to the presence of the Fenton reaction. Under normoxic conditions, the FPTM + X‐ray group displayed an enhanced capacity for ROS generation compared to that under hypoxic conditions. This can be attributed to the fact that hypoxia impeded the RT‐RDT effect of FPTM, leading to the decrease of ROS generation (Figure S11, Supporting Information). Specifically, FPTM‐LP enhanced ROS levels through three main mechanisms: 1) Pt effectively absorbed radiation energy to generate •OH, while transferring X‐ray energy to TCPP, which excited the generation of ^1^O_2_. 2) The released LND interfered with mitochondrial complexes I and II, inhibiting OXPHOS, which reduced the OCR and alleviated hypoxia, consequently increasing ROS generation. 3) Fe^2+^ reacted with excessive endogenous H_2_O_2_ through the Fenton reaction to generate •OH.

### 2.4 In Vitro Radiosensitizing Effects of FPTM‐LP

**Figure 4:**
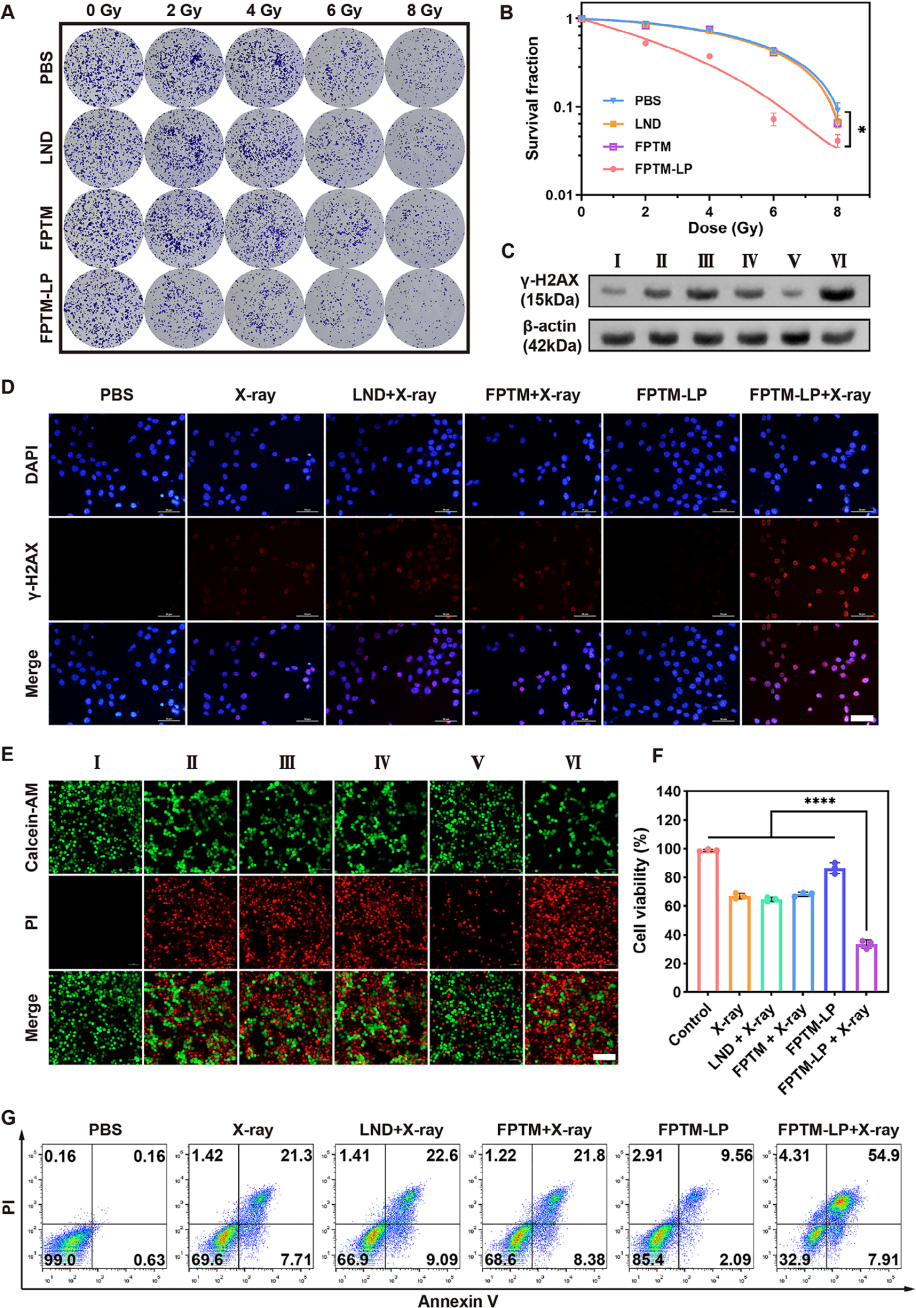
Enhanced RT‐RDT effect in vitro under hypoxic conditions. A) The results of the colony formation assay for each group. B) Survival fraction curves. C) Western blot results of γ‐H2AX expression. D) CLSM images of immunostaining for γ‐H2AX (Scale bar: 50 µm). E) CLSM images of calcein‐AM/PI staining (I: PBS; II: X‐ray; III: LND + X‐ray; IV: FPTM + X‐ray; V: FPTM‐LP; VI: FPTM‐LP + X‐ray. Scale bar: 100 µm). F) CCK‐8 assay results with different treatments. G) Flow cytometry results of apoptosis with different treatments. *n* = 3 for each sample. Data were expressed as mean ± SD. Differences were analyzed using one‐way ANOVA (**** for *p* < 0.0001, *** for *p* < 0.001, ** for *p* < 0.01, and * for *p* < 0.05).

To investigate the radiosensitizing effect of FPTM‐LP for 4T1 cells under hypoxic conditions, a colony formation assay was performed. No significant growth inhibition of tumor cells was found in either the LND group or the FPTM group compared to the control group at the same radiation dose. In contrast, the FPTM‐LP group demonstrated a marked inhibition of clone formation (**Figure** **4**A). The survival fraction curve showed that the clone survival rate of 4T1 cells gradually decreased with increasing radiation doses (Figure 4B). Notably, at a radiation dose of 6 Gy, FPTM‐LP exhibited significant difference in radiation‐induced inhibition of colony formation compared to the other groups. Although increasing the radiation dose can enhance the killing effect on 4T1 cells, an excessively high dose may also lead to severe damage to normal tissues, triggering side effects. Therefore, we selected 6 Gy as the optimal irradiation dose for subsequent experiments. Mechanistically, the enhanced efficacy of the combined therapy may be attributed to increasing DNA damage. Significant upregulation of γ‐H2AX was observed by western blot analysis (Figure 4C and Figure S12, Supporting Information) after treatment with FPTM‐LP under X‐ray irradiation. Consistent with these findings, immunofluorescence staining of γ‐H2AX revealed bright red fluorescence in all X‐ray‐exposed groups, with the FPTM‐LP + X‐ray group displaying the strongest fluorescence signal (Figure 4D). Furthermore, the decrease in intracellular GSH levels further suggested that exogenous Fe^3^⁺ derived from FPTM‐LP participated in GSH consumption through a GSH‐mediated reduction process converting Fe^3^⁺ to Fe^2^⁺ (Figure S13, Supporting Information). Moreover, the depletion of GSH shifted the intracellular redox balance toward oxidative stress and inhibited DNA damage repair. Additionally, a live/dead cell staining assay demonstrated that FPTM‐LP combined with RT significantly decreased the survival rate of 4T1 cells under hypoxic conditions (Figure 4E), which was further supported by the CCK‐8 cell viability assay (Figure 4F). However, the FPTM+X‐ray group exhibited disappointing results. In contrast, the FPTM+X‐ray group demonstrated a cell‐killing ability comparable to that of the FPTM‐LP+X‐ray group under normoxic conditions (Figure S14, Supporting Information). This finding underscores the critical importance of O_2_ economization for achieving the RT‐RDT effect in hypoxic TME. Flow cytometry results indicated that the combined application of FPTM‐LP and RT caused the highest level of apoptosis, with an apoptosis rate more than twice that of X‐ray monotherapy (Figure 4G). This result was ascribed to the disruption of mitochondrial membrane integrity, leading to the release of apoptosis‐inducing factors and initiating the mitochondrial‐mediated apoptotic cascade. Additionally, DNA damage caused by excessive ROS will also trigger apoptosis.^[^ ^28^ ^]^ In summary, these data strongly suggested that, as an O_2_ economizer and ROS amplifier, FPTM‐LP significantly improved RT‐RDT efficacy.

### 2.5 Biodistribution and Hypoxia Imaging Property of FPTM‐LP In Vivo

**Figure 5:**
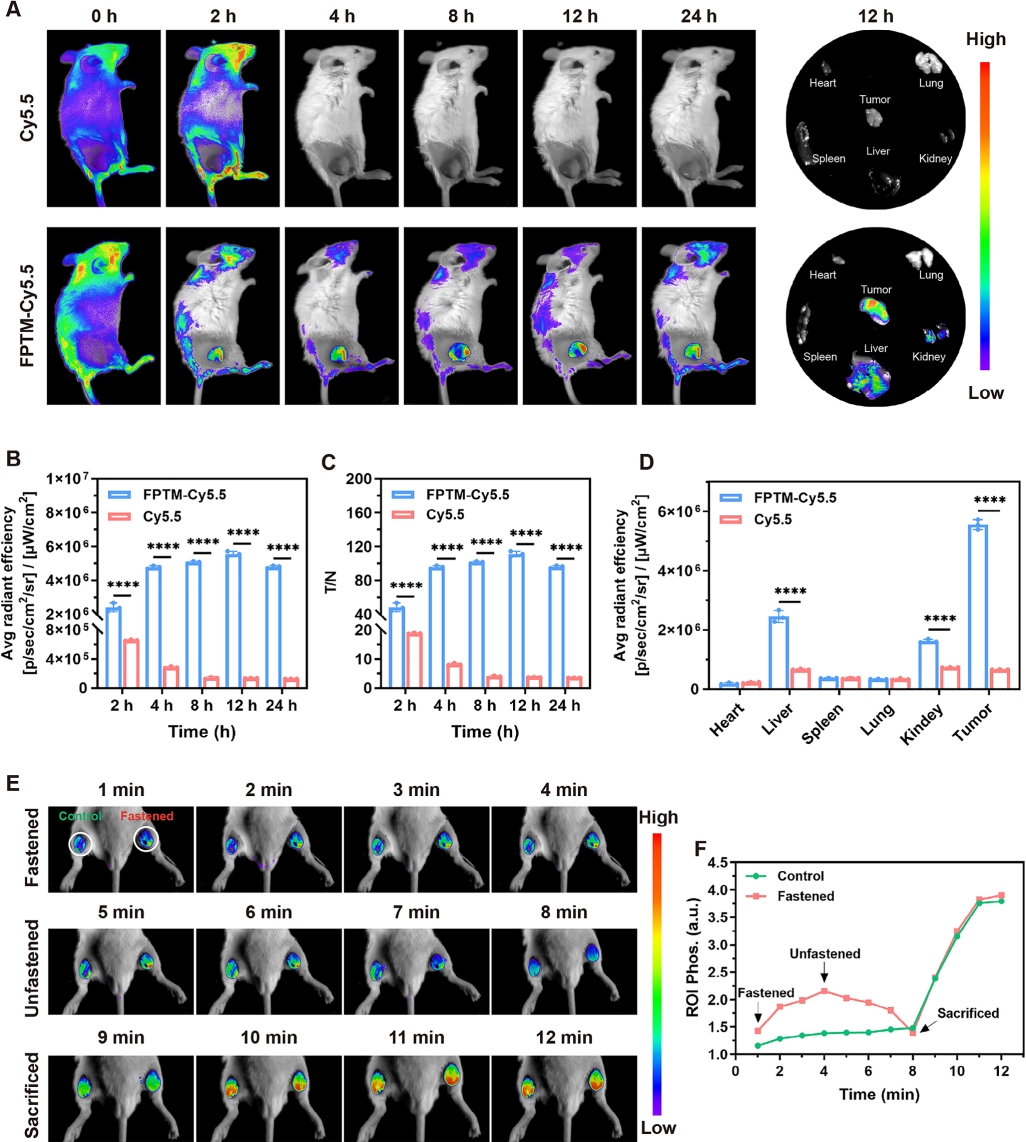
Biodistribution imaging and real‐time hypoxia imaging in vivo. A) Biodistribution images of FPTM‐Cy5.5 at different time points, and ex vivo fluorescence images of tumors and main organs at 12 h after injection. B) Mean fluorescence intensity of tumors at specific time points. C) The ratio of average fluorescence intensity between tumor and non‐tumor tissues at specific time points. D) Mean fluorescence intensity of tumors and main organs at 12 h after injection. E) Real‐time hypoxia imaging of 4T1 tumor‐bearing mice. F) Quantitative luminescence intensity analysis. *n* = 3 for each sample. Data were expressed as mean ± SD. Differences were analyzed using an unpaired Student's *t* test. (**** for *p* < 0.0001, *** for *p* < 0.001, ** for *p* < 0.01, and * for *p* < 0.05).

4T1 tumor bearing mice were injected with free cyanine dye Cy5.5 and Cy5.5‐labeled FPTM (FPTM‐Cy5.5) via tail vein. Afterwards, the in vivo biodistribution of nanoparticles was monitored by fluorescence imaging at specific time points. As displayed in **Figure** **5**A, the accumulation of FPTM‐Cy5.5 at the tumor site was detectable at 2 h and reached fluorescence signal peak at 12 h. At the peak of fluorescence intensity, the average intensity at the tumor site was 2.3‐fold higher than that at 2 h (Figure 5B), and the fluorescence intensity ratio between tumor and non‐tumor tissues reached maximum (Figure 5C). Excitingly, the fluorescence intensity remained at a relatively high level even at 24 h. However, free Cy5.5 was rapidly cleared from the body and did not accumulate at the tumor site. A marked increase of fluorescence intensity in tumors compared to all harvested organs was revealed by ex vivo fluorescence imaging after injection of FPTM‐Cy5.5 (Figure 5D). Notably, the noticeable fluorescence intensity in the liver and kidneys indicated that FPTM‐Cy5.5 and its degradation product may be metabolized through these organs. To explore the in vivo metabolism of FPTM‐LP, quantitative analysis of Pt element in tumor, liver, and kidneys at different time points was further measured by inductively coupled plasma optical emission spectrometry (ICP‐OES). The results revealed that the Pt element in tumor tissues peaked at 12 h and then gradually decreased, accompanied by an increased accumulation in the liver and kidneys (Figure S15, Supporting Information). This confirmed the hypothesis that FPTM‐LP was metabolized via the hepatic and renal pathways. The prolonged blood circulation time and enhanced permeation and retention (EPR) effect allow FPTM‐LP to accumulate at the tumor site for an extended period, providing the possibility for in vivo treatment.

Encouraged by the O_2_‐sensing ability in vitro, the hypoxia imaging capability of FPTM in vivo was further verified. Local O₂ concentrations were modulated by ligating blood vessels, thereby restricting blood flow to the tumors. Within 0 to 4 min after blood flow restriction in the right hind leg, luminescence intensity of tumor on the right side gradually increased (Figure 5E). Following the release of the ligation, luminescence intensity gradually decreased over the next 5 to 8 min. The left hind leg, which served as the control, showed no significant changes in luminescence intensity throughout the process. After euthanasia, the tumors in both hind legs of the mice exhibited a significant increase in luminescence intensity. Additionally, similar changes in luminescence intensity were identified in the quantitative analysis (Figure 5F). The above data fully confirmed that changes in O_2_ levels can be rapidly, accurately, and reversibly reported by FPTM in vivo.

### 2.6 Evaluation of Hypoxia Alleviation Guided by Luminescence Imaging and [^18^F]‐FMISO PET Imaging

**Figure 6:**
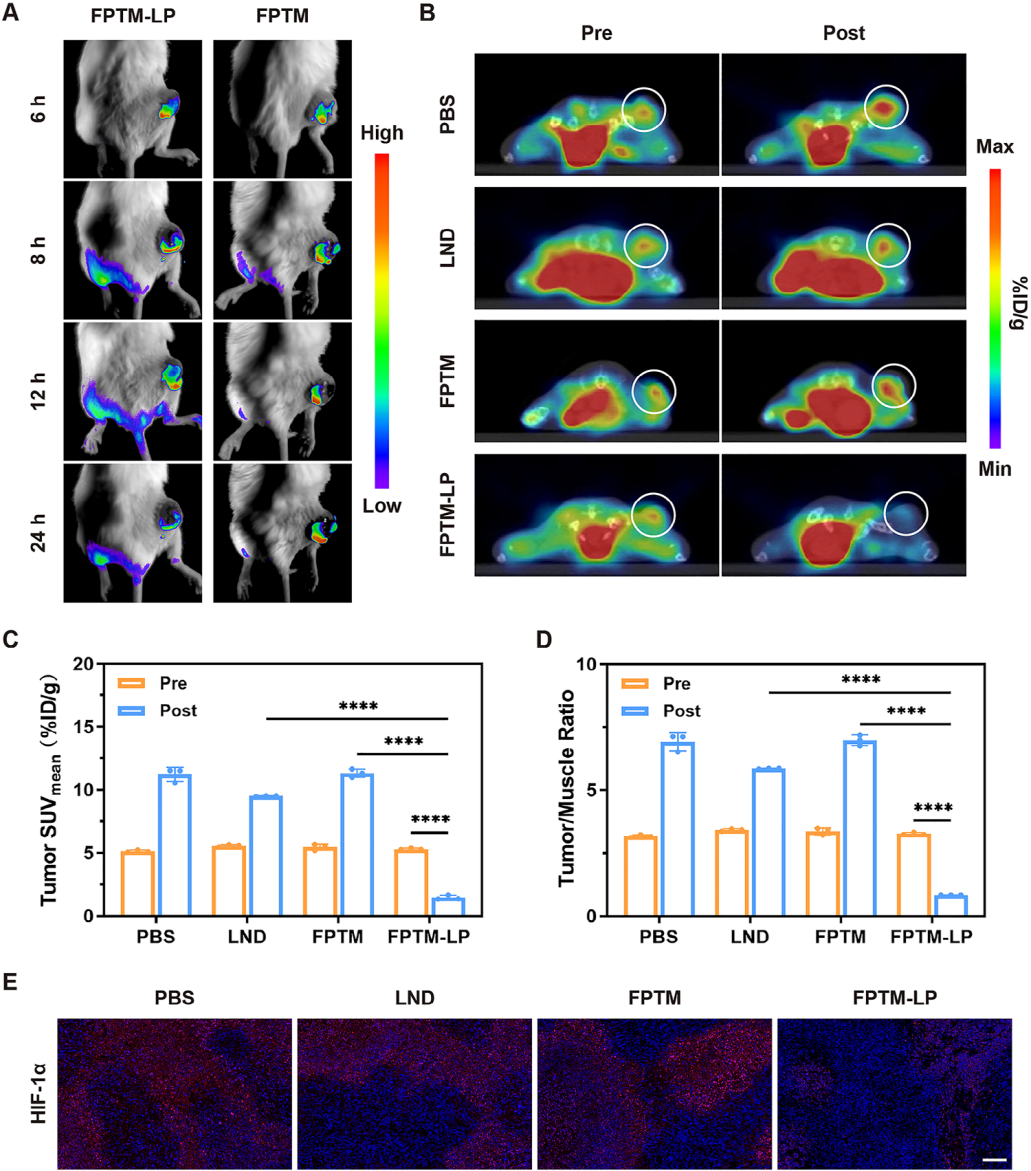
Efficacy of FPTM‐LP in alleviating tumor hypoxia in vivo. A) In vivo luminescence images of 4T1 tumor‐bearing mice with different treatments. B) PET/CT images of mice after injection with [^18^F]‐FMISO (the circled areas indicate the tumors). C) SUV_mean_ of [^18^F]‐FMISO imaging pre and post different treatments. D) T/M ratio of [^18^F]‐FMISO imaging pre and post different treatments. E) HIF‐1α staining images of tumors (Scale bar: 50 µm). *n* = 3 for each group. Data were expressed as mean ± SD. Differences were analyzed using one‐way ANOVA (**** for *p* < 0.0001, *** for *p* < 0.001, ** for *p* < 0.01, and * for *p* < 0.05).

In light of the promising hypoxia alleviation effect of FPTM‐LP at the cellular level, the hypoxia alleviation capability in vivo was further studied. First, tumor hypoxia was investigated utilizing the self‐reporting luminescence imaging capability of FPTM. The luminescence intensity of the tumor gradually decreased 12 h post‐injection and showed a significant difference at 24 h, indicating that FPTM‐LP significantly reversed tumor hypoxia in vivo (**Figure** **6**A). To further validate this result, ^18^F‐fluoromisonidazole ([^18^F]‐FMISO) micro‐PET/CT, the most prevalent method for hypoxia imaging in clinical practice, was conducted.^[^ ^29^ ^]^ The lipophilic [^18^F]‐FMISO passively diffused into tumor tissues and was then reduced by nitroreductases. In normoxic cells, the reduced product of [^18^F]‐FMISO was oxidized to its original compound and escaped from the cells. However, in hypoxic cells, the reoxidation process of [^18^F]‐FMISO product was limited, which led to irreversible binding to biomolecules and significant retention of probe, allowing for quantitative monitoring of hypoxia levels.^[^ ^30^ ^]^ Micro‐PET/CT scans were carried out 40 min after the administration of 3.7 MBq of [^18^F]‐FMISO. To determine the tracer uptake in the tumor area, regions of interest (ROIs) were delineated and quantified, and the mean standardized uptake value (SUV_mean_) was calculated. The PET/CT images revealed that the most significant reduction in SUV_mean_ occurred in the FPTM‐LP group (Figure 6B), with a decrease of 72.60% compared to pre‐treatment levels (Figure 6C). Additionally, the tumor‐to‐muscle (T/M) uptake ratio decreased to 0.84 ± 0.01 (Figure 6D). It is noteworthy that LND also controlled the aggravation of tumor hypoxia to a certain extent, but its effect was extremely limited due to its low bioavailability. Besides, HIF‐1α immunofluorescence staining of tumor tissue sections was conducted after treatment. HIF‐1α expression was evident in the PBS group, whereas negligible expression was observed in the FPTM‐LP group (Figure 6E and Figure S16, Supporting Information). These findings were consistent with the western blot analysis and hypoxia imaging results.

### 2.7 Monitoring of Glucose Metabolism in Tumors by [^18^F]‐FDG PET Imaging

**Figure 7:**
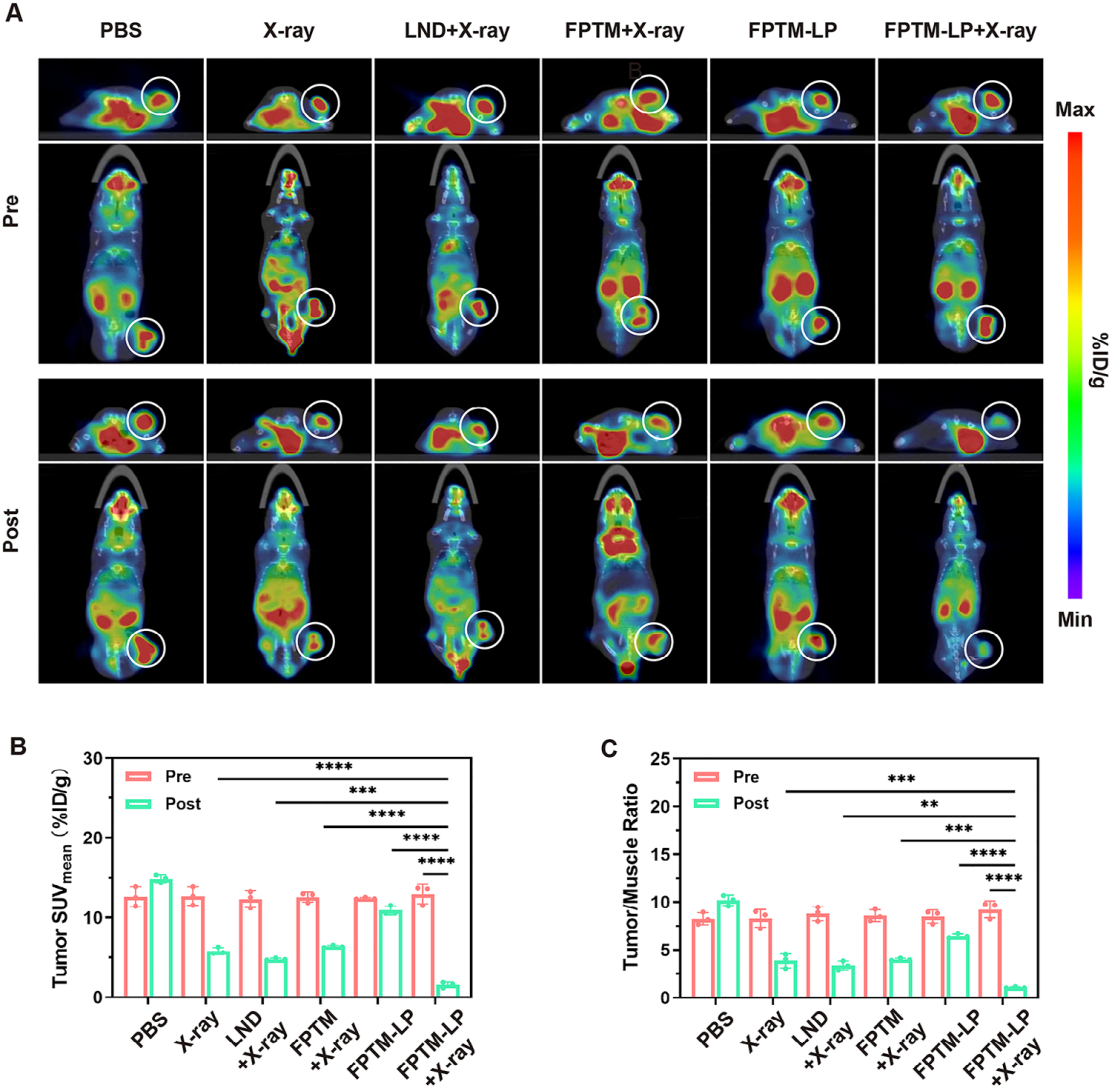
[^18^F]‐FDG PET/CT monitoring of glucose metabolism of 4T1 tumors. A) PET/CT images of mice after injection with [^18^F]‐FDG (the circled areas indicate the tumors). B) SUV_mean_ of [^18^F]‐FDG imaging pre and post different treatments. C) T/M ratio of [^18^F]‐FDG imaging pre and post different treatments. *n* = 3 for each sample. Data were expressed as mean ± SD. Differences were analyzed using one‐way ANOVA (**** for *p* < 0.0001, *** for *p* < 0.001, ** for *p* < 0.01, and * for *p* < 0.05).

Considering that FPTM‐LP exhibited the high‐efficiency cytotoxicity in vitro and the high tumor uptake capacity in vivo, we established subcutaneous 4T1 breast cancer mouse models to validate the in vivo antitumor effect of FPTM‐LP. At the molecular level, the early treatment response of malignant tumors is initially manifested as a variation in metabolism. Therefore, the change of glucose metabolism precedes the change of tumor size. β‐2‐[^18^F]‐Fluoro‐2‐deoxy‐D‐glucose ([^18^F]‐FDG) metabolic imaging of tumors provides information on glucose metabolism, enabling the early detection of metabolic changes during treatment. After the tumor volume reached approximately 100 mm^3^, the mice were randomly divided into six groups. Twelve hours after injection of PBS, LND, FPTM, or FPTM‐LP via tail vein, the mice received a single dose of 6 Gy X‐ray irradiation. Before and after treatment, 3.7 MBq of [^18^F]‐FDG was administered via tail vein, and micro‐PET/CT scans were performed at 40 min post‐injection. As shown in **Figure** **7**A, X‐ray irradiation caused a decrease in glucose uptake of tumors, with the most significant reduction occurring in the FPTM‐LP + X‐ray group. The SUV_mean_ of tumors treated with FPTM‐LP and X‐ray was 1.56 ± 0.35 %ID g^−1^, representing an 87.94% decrease compared to pre‐treatment (Figure 7B). Additionally, the T/M uptake ratio decreased to 1.08 ± 0.08 (Figure 7C). The obvious metabolism suppression for tumors in the early stage raised our expectations for the long‐term therapeutic efficacy of FPTM‐LP.

### 2.8 FPTM‐LP Enhanced the Effectiveness of RT‐RDT In Vivo

**Figure 8:**
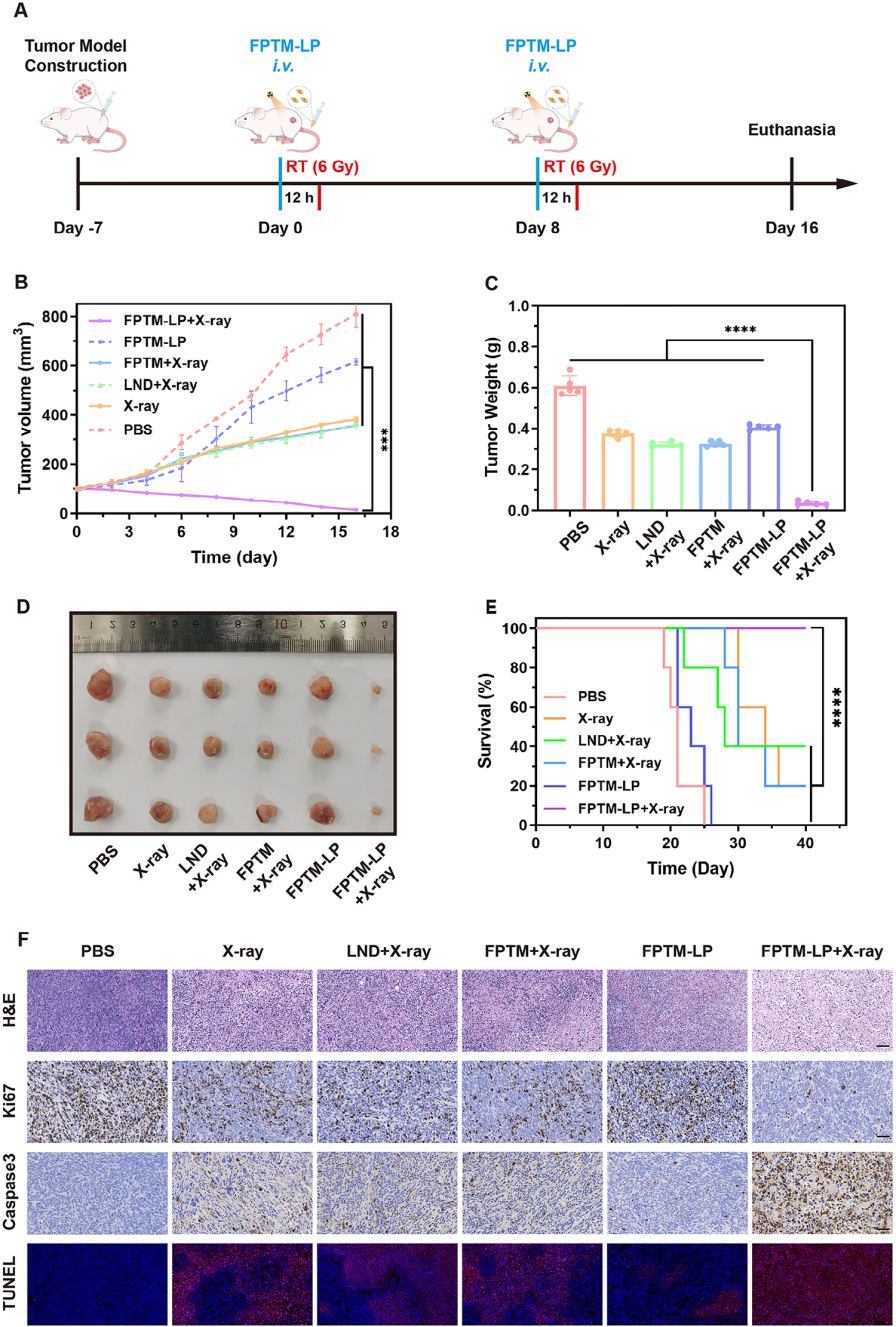
Antitumor effect of FPTM‐LP in vivo. A) Flowchart of tumor treatment. B) Tumor volume growth curves (*n* = 5). C) Average tumor weights of each group at the end of treatment (*n* = 5). D) Photographs of tumors after treatment. E) Survival curves of each group (*n* = 5). F) H&E, Ki67, Caspase3, and TUNEL staining images of tumors (Scale bar: 50 µm). Data were expressed as mean ± SD. Differences were analyzed using one‐way ANOVA or the log‐rank test. (**** for *p* < 0.0001, *** for *p* < 0.001, ** for *p* < 0.01, and * for *p* < 0.05).

The treatment procedure for 4T1 tumor‐bearing mice was shown in **Figure** **8**A. Based on the records of tumor volume over a 16‐day period (Figure S17, Supporting Information), a slight tumor suppression effect was observed in the FPTM‐LP group without X‐ray irradiation compared to the PBS group. In contrast, the X‐ray group exhibited a certain degree of tumor growth inhibition. Although tumor growth in the LND + X‐ray group was restricted, the therapeutic outcome was insufficient. Encouragingly, the combination of FPTM‐LP and X‐ray irradiation induced the strongest antitumor effect, with tumor volume reducing 85.8% ± 1.9% (Figure 8B). At the study endpoint, mice were euthanized, and tumors were weighed and photographed. As depicted in Figure 6C,D and Table S1 (Supporting Information), the FPTM‐LP + X‐ray group showed the lowest tumor weight, which was consistent with the time‐dependent tumor volume curve. Notably, the survival rate of the mice in the FPTM‐LP + X‐ray group increased significantly (Figure 8E and Table S2, Supporting Information). Subsequently, the hematoxylin‐eosin (H&E) staining results demonstrated that, in the FPTM‐LP + X‐ray group, the nuclei of tumor cells were condensed and fragmented, appearing blue‐black (Figure 8F). Membrane rupture and cytoplasm extrusion led to areas with homogeneous, amorphous, and light red granular regions, indicating a significant increase in cell necrosis and apoptosis. Afterwards, Caspase‐3 staining, TdT‐mediated dUTP Nick‐End Labeling (TUNEL) staining and Ki67 staining was employed to study the apoptosis and proliferation of tumors. Unsurprisingly, the FPTM‐LP + X‐ray group exhibited the lowest number of Ki67‐positive cells, which is an indicator of cell proliferation. Semiquantitative assessment of Ki67‐positive cells showed that the cellular proliferation rate in this group was only 4.49% ± 0.84%, significantly lower than other groups (Figure S18, Supporting Information). Furthermore, the positive rate of the apoptosis marker Caspase‐3 was highest in the FPTM‐LP + X‐ray group (stained dark brown), with an approximately 2.5‐fold increase in the apoptosis rate compared to the X‐ray monotherapy group (Figure S19, Supporting Information). The TUNEL staining results (red fluorescence) further confirmed that this group had the highest level of cellular apoptosis, supported by semi‐quantitative fluorescence analysis of TUNEL staining (Figure S20, Supporting Information). All these data indicated that FPTM‐LP with X‐ray irradiation exerted a remarkable antitumor effect through O_2_‐economized enhanced RT‐RDT.

### 2.9 Biosafety Assessment of FPTM‐LP

**Figure 9:**
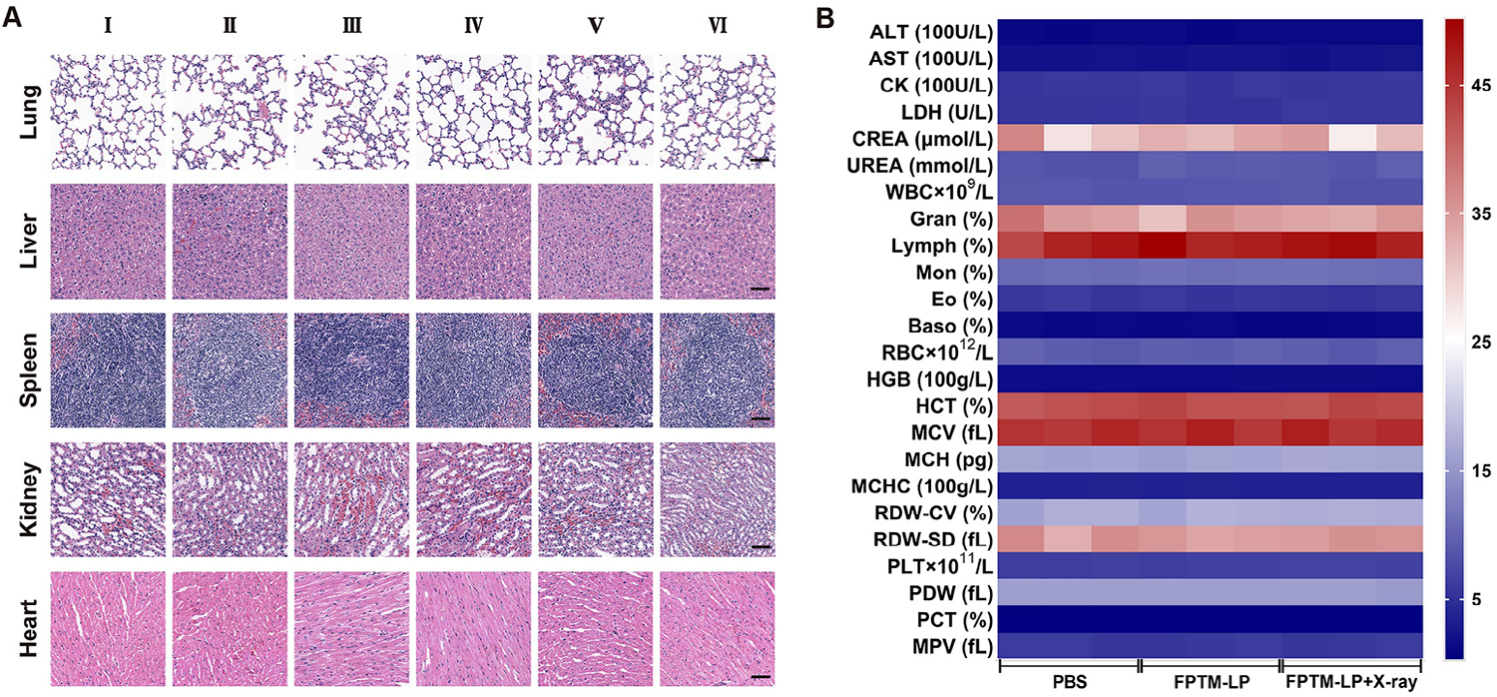
Biological safety evaluation. A) H&E images of the major organs (lungs, liver, spleen, kidneys and heart) after treatment (I: PBS; II: X‐ray; III: LND + X‐ray; IV: FPTM + X‐ray; V: FPTM‐LP; VI: FPTM‐LP + X‐ray. Scale bar: 50 µm). B) Blood biochemistry and blood routine analysis after treatment.

The in vivo biosafety of nanomaterials is crucial for bioapplications. Overall, the body weight recorded every two days during the treatment period remained relatively stable, with negligible changes observed (Figure S21, Supporting Information). No significant histopathological damage was found in the lungs, liver, spleen, kidneys and heart (**Figure** **9**A). Additionally, there was no apparent change in the biochemical indicator, including aspartate aminotransferase (AST), alanine aminotransferase (ALT), creatinine (CREA), and urea (UREA), which suggested the normal physiological functions of the liver and kidneys. Blood routine analysis revealed that the primary hematological parameters remained within the reference ranges after treatment (Figure 9B and Table S3, Supporting Information). All available evidence demonstrated the excellent biosafety of FPTM‐LP as an O_2_ economizer and ROS amplifier for enhanced RT‐RDT.

## 3 Conclusion

In summary, we constructed a weak acidity and GSH‐responsive nano‐radiosensitizer (FPTM‐LP) to address the challenges of insufficient energy deposition, tumor hypoxia, and low absorption coefficients, paving the way for effective RT‐RDT. The high‐Z metal‐porphyrin (Pt(II)TCPP) can effectively absorb X‐ray and product •OH and ^1^O_2_ for RT‐RDT. The GSH‐responsively released LND and Fe^2+^ were subsequently applied for reducing O_2_ consumption and promoting ROS generation. Besides, FPTM‐LP enabled near‐infrared luminescence imaging to provide a self‐monitoring of O_2_ saturation, showing a real‐time tumor hypoxia relief of FPTM‐LP in vivo. [^18^F]‐FMISO PET imaging also validated the effective reversal of tumor hypoxia treated with FPTM‐LP. Both in vitro and in vivo experiments revealed that the FPTM‐LP greatly enhanced RT‐RDT efficacy under X‐ray irradiation, resulting in the most efficient suppression effect observed in breast tumor‐bearing mice. Encouragingly, FPTM‐LP demonstrates excellent biosafety, providing a new strategy for treating radiation‐resistant breast cancer.

## 4 Experimental Section

### Materials and Reagents

Pt(II) meso‐tetra(4‐carboxyphenyl) porphine (Pt(II) TCPP), iron chloride hexahydrate (FeCl_3_·6H_2_O), and anhydrous ethanol (C_2_H_6_O) were obtained from Aladdin Holdings Group Co., Ltd (China). N,N‐Dimethylformamide (DMF), benzoic acid (C_7_H_6_O_2_), disodium terephthalate (TA), lonidamine (LND), cyanine3 (Cy3), cyanine5.5 (Cy5.5), dimethyl sulfoxide (DMSO), and poly (ethylene glycol)‐2000 (PEG‐2000) were purchased from Shanghai Macklin Biochemical Technology Co., Ltd (China). PBS, penicillin‐streptomycin double antibody, trypsin, DMEM, and FBS were obtained from Gibco Invitrogen (USA). *_L_*‐glutathione (GSH) reduction and calcein‐AM/PI assay kit were obtained from Solarbio (China). CCK‐8 assay kit, DNA damage assay kit by γ‐H2AX immunofluorescence, DAPI, DCFH‐DA, SOSG, JC‐1 and ANNEXIN‐FITC/PI double dye kit were obtained from Beyotime Biotechnology (Shanghai China). Antibodies (Anti‐β actin, Anti‐GAPDH, γ‐H2AX and HIF‐1α) were provided from Beijing 4A Biotech Co., Ltd (China). [^18^F]‐FDG was produced according to standard methods using the coincidence [^18^F]‐FDG synthesis module (Synthera V2, IBA). [^18^F]‐FMISO was produced followed the procedure previously described.^[^ ^31^ ^]^ Transwell chamber and other cell culture plastic products purchased from NEST Biotechnology (Wuxi, China).

### Characterization

TEM images, element mapping and SAED patterns were obtained with a FEI Talos F200X G2 transmission electron microscope. XRD was detected using a Rigaku D/MAX‐2600 X‐ray diffractometer. XPS measurements were performed using a Thermo Scientific K‐Alpha spectroscopy. DLS and zeta potential were measured with a Malvern Zetasizer Nano ZS90 instrument. UV‐vis absorption spectra of FPTM‐LP were conducted by a PE lambda 750 spectrophotometer. Luminescence emission spectra and phosphorescence lifetime were carried out with an Edinburgh FLS980 spectrophotometer. ESR spectra were recorded using a Bruker EMXplus spectrometer. CLSM images were acquired using a Zeiss LSM 880 microscopy. In vivo and ex vivo fluorescence imaging were performed by an IVIS Lumina imaging system. ICP‐OES was performed by an PerkinElmer 8300 inductively coupled plasma optical emission spectrometer. Flow cytometric analysis was conducted by BD FACS Aria II. Micro‐PET/CT images were recorded using a micro‐PET/CT (Madic Technology Co., Ltd, Shandong, China).

### Preparation of FPTM and FPTM‐LP

FeCl₃·6H_2_O (30 mg)and benzoic acid (280 mg) were added into DMF (6 mL) and the mixture was stirred for 10 min. Then Pt(II)TCPP in DMF (5 mL, 2.5 mg mL^−1^) was slowly added into the above solution and the mixture was further stirred at 120 °C for 24 h. Finally, the mixture was centrifuged (10 000 rpm, 10 min) and the precipitate was washed three times with DMF, yielding an orange‐red product, FPTM. The FPTM utilized in both cellular and animal experiments had been modified with PEG‐2000. FPTM was mixed with lonidamine and stirred overnight. Subsequently, the mixture was centrifuged (10 000 rpm, 10 min) and the precipitate was washed three times with DMF. Then the product was resuspended in an aqueous solution of PEG‐2000 and stirred overnight. Finally, the product was washed again to obtain the purified FPTM‐LND‐PEG (FPTM‐LP).

### Extracellular O_2_‐Sensing Ability

The gas mixtures of N_2_ and O_2_ with different O_2_ concentrations (1%, 10%, 21%, 50%, or 100%) were introduced into an FPTM solution (20 mg mL^−1^). After 2 h of treatment, the emission spectra and phosphorescence lifetime were recorded and analyzed using an Edinburgh FLS980 spectrophotometer.

### GSH/pH Response

Different concentrations of GSH and PBS solutions with different pH were added to a FPTM‐LP solution (0.2 mg mL^−1^), and the hydrodynamic diameter, morphological changes and UV–vis spectra were analyzed after incubation. Furthermore, changes in Fe^2+^ content and drug release profiles were monitored at various time intervals during incubation.

### Extracellular ^•^OH and ^1^O_2_ Generation

^1^O_2_ generation was detected by a SOSG fluorescent probe. Briefly, PBS and FPTM (100 µg mL^−1^) were pre‐mixed with SOSG (5 µM) in 2% aqueous methanol. The mixture was subsequently irradiated with X‐ray at varying doses of 0, 2, 4, 6, or 8 Gy, and the fluorescence spectra were recorded with a fluorescence spectrometer. For the detection of •OH, the specific indicator disodium terephthalate (TA) was employed, which captures •OH to form the fluorescent product disodium 2‐hydroxy terephthalate (TAOH).^[^ ^32^ ^]^ To verify the enhanced RT effect, PBS, FPTM (100 µg mL^−1^), and TA (5 mm) were pre‐mixed and subsequently irradiated with X‐ray at varying doses of 0, 2, 4, 6, or 8 Gy. Separately, to validate the Fenton reaction, FPTM (100 µg mL^−1^), TA (5 mm), GSH (10 mm), and H_2_O_2_ were mixed together. The fluorescence spectra of TAOH were recorded with a fluorescence spectrometer.

### Cells and Animals

4T1 cells and L929 cells were gained from the American Type Culture Collection (USA). Cells were incubated in DMEM medium (10% FBS) at 37 °C with 5% CO_2_. Female Balb/c mice, aged 5–6 weeks were obtained from Beijing HFK Bio‐Technology Co., Ltd (China). All animal procedures were conducted following the guidelines and regulations established by the Institutional Animal Care and Use Committee at School of Public Health of Jilin University (SY:2024‐10‐008).

### Cellular Uptake

FPTM (2 mg) were dispersed in DMF, followed by the dissolution of Cy3 (2 mg) under sonication. Subsequently, the mixture was centrifuged (10 000 rpm, 10 min) and the precipitate was washed three times with DMF. Then the product was resuspended in an aqueous solution of PEG‐2000 and stirred overnight. Finally, the product was washed again to obtain the purified FPTM‐Cy3. 4T1 cells (5×10^4^ cells per well) were plated in confocal dishes and incubated overnight. FPTM‐Cy3 and free Cy3 (100 µg mL^−1^) in DMEM were then added to the confocal dishes and co‐cultured for 2, 4, 8 or 12 h. After staining with DAPI, cellular uptake was observed using CLSM.

### Intracellular ATP and GSH Measurements

4T1 cells (1×10^4^ cells per well) were inoculated in a 96‐well plate and incubated overnight. The cells were pretreated with PBS, LND, FPTM or FPTM‐LP (100 µg mL^−1^). After 12 h of cultivation, the intracellular ATP and GSH levels were measured using the ATP assay kit and GSH assay kit.

### Intracellular O_2_‐Sensing Ability

4T1 cells were treated with FPTM (100 µg mL^−1^) at different O_2_ concentrations (1%, 10%, 21%, or 50% O_2_) for 24 h. Alternatively, 4T1 cells were treated with FPTM, LND + FPTM, and FPTM‐LP (100 µg mL^−1^) under hypoxic conditions for 24 h. Luminescence emission was imaged using CLSM (*λ*ex = 405 nm, *λ*em = 760 nm).

### Cell Viability Assay

4T1 cells (1×10^4^ cells per well) were inoculated in a 96‐well plate and incubated overnight. To evaluate the biocompatibility of the nanoparticles, the cells were treated with FPTM‐LP (0, 6.25, 12.5, 25, 50, 100 µg mL^−1^) for 12 h. To detect the radiosensitization effect of FPTM‐LP, cells were pretreated with PBS, LND, FPTM or FPTM‐LP (100 µg mL^−1^) for 12 h under hypoxic conditions, with or without X‐ray irradiation (1 Gy min^−1^, 6 min). After 24 h of cultivation, 100 µL of DMEM containing CCK8 (10 µL) was added to the cells and incubated in the dark. The absorbance values of each well were detected with a microplate reader.

### Colony Formation Assay

4T1 cells (1000 cells per well) were plated in a six‐well plate and incubated overnight. Subsequently, the cells were pretreated with PBS, LND, FPTM or FPTM‐LP (100 µg mL^−1^) for 12 h under hypoxic conditions before being irradiated with 0, 2, 4, or 6 Gy, respectively. The cells were then cultured in an incubator for 2 weeks. Finally, the cell colonies were stained, imaged, and the number of colonies (≥50 cells) were enumerated. Survival curves were then plotted and analyzed. Survival Fraction = colonies formed after treatment / cells seeded × 100%.

### Live/Dead Staining Assay

4T1 cells (1×10^5^ cells per well) were plated in confocal dishes and incubated overnight. The cells were pretreated with PBS, LND, FPTM or FPTM‐LP (100 µg mL^−1^) for 12 h, with or without X‐ray irradiation (1 Gy min^−1^, 6 min). After 24 h of cultivation, the cells were stained with 500 µL Calcein‐AM (10 µm) and PI (10 µm) and incubated at 37 °C in the dark for 30 min. The cell viability was observed using CLSM.

### Flow Cytometry Assay of Apoptosis

4T1 cells (2×10^5^ cells per well) were plated in a six‐well plate and cultured under hypoxic conditions overnight. The cells were pretreated with PBS, LND, FPTM or FPTM‐LP (100 µg mL^−1^) for 12 h, with or without X‐ray irradiation (1 Gy min^−1^, 6 min). After 24 h of cultivation, the cells were stained with ANNEXIN‐FITC/PI in the dark at 37 °C for 15 min. Finally, cell apoptosis was analyzed by flow cytometry.

### Intracellular ROS Generation

4T1 cells (1×10^5^ cells per well) were plated in confocal dishes and incubated overnight. The cells were pretreated with PBS, LND, FPTM or FPTM‐LP (100 µg mL^−1^) for 12 h, with or without X‐ray irradiation (1 Gy min^−1^, 6 min). After 24 h of cultivation, the cells were treated with 500 µL DCFH‐DA (10 µM) at 37 °C in the dark for 20 min. The ROS generation was analyzed by flow cytometry and imaged using CLSM.

### Western Blot Assay

4T1 cells (2×10^5^ cells per well) were plated in a six‐well plate and incubated under hypoxic conditions overnight. The cells were pretreated with PBS, LND, FPTM or FPTM‐LP (100 µg mL^−1^) for 12 h, with or without X‐ray irradiation (1 Gy min^−1^, 6 min). After 24 h of cultivation, the cells were digested, centrifuged, washed, and lysed to extract the protein solution, and the protein content was detected using the Bradford protein concentration kit. Extracted proteins (γ‐H2AX and HIF‐α) were separated by precast gel and transferred to PVDF membrane. The transferred PVDF membranes were then sealed with immunostaining blocking buffer for 1 hour and incubated overnight at 4 °C with the corresponding antibodies. Samples were then shaken with secondary antibody at 4 °C for 1 hour. Finally, protein bands were visualized by adding enhanced chemiluminescence (ECL) developer dropwise using a Bio‐Rad ChemiDoc XR+ imaging system.

### Mitochondrial Membrane Potential Detection

4T1 cells (1×10^5^ cells per well) were plated in confocal dishes and incubated overnight. The cells were pretreated with PBS, LND, FPTM or FPTM‐LP (100 µg mL^−1^). After 12 h of cultivation, the cells were stained with JC‐1 reagent for 30 min. Changes in mitochondrial membrane potential were imaged using CLSM.

### γ‐H2AX Immunofluorescence Assay

4T1 cells (1×10^5^ cells per well) were plated in confocal dishes and incubated under hypoxic conditions overnight. The cells were pretreated with PBS, LND, FPTM or FPTM‐LP (100 µg mL^−1^) for 12 h, with or without X‐ray irradiation (1 Gy min^−1^, 6 min). After 24 h of cultivation, the cells were fixed using 4% paraformaldehyde (15 min), permeated with Triton‐X 100, blocked with 1% BSA buffer (1 h), and then incubated with γ‐H2AX antibody at 4 °C overnight. Then, the cells were shaken with secondary antibody at room temperature for 2 h. Finally, DNA damage was imaged using CLSM.

### Construction of the Subcutaneous Tumor Model

4T1 cell suspension (1×10^6^ cells mL^−1^, 100 µL) was injected into the dorsal subcutaneous tissue of female Balb/c mice aged 5–6 weeks.

### Biodistribution Imaging In Vivo

The preparation method for FPTM‐Cy5.5 is the same as that for FPTM‐Cy3. FPTM‐Cy5.5 was injected via the tail vein. Biodistribution images were acquired at specified time points using IVIS imaging system.

### Retention and Metabolism of FPTM‐LP In Vivo

At 2, 6, 12, 24, and 72 h after tail vein injection of FPTM‐LP, the mice were euthanized and the tumors, livers, and kidneys were collected. The Pt(II) content in these samples was analyzed using ICP‐OES.

### Hypoxia Imaging In Vivo

Both hind legs of mice were intratumorally injected with FPTM (200 µL, 1 µg mL^−1^). After 30 min, the left hind leg was designated as a control, while the right hind leg was ligated for 4 min, then unfastened for another 4 min. The mice were subsequently euthanized. Throughout this period, the IVIS imaging system was used to monitor changes in luminescence signal intensity (*λ*ex = 405 nm, *λ*em = 760 nm).

### Micro‐PET/CT Imaging In Vivo

After inhalation anesthesia, [^18^F]‐FDG or [^18^F]‐FMISO (3.7 MBq) were injected into each group of mice via tail vein. Subsequently, the mice were scanned using a micro‐PET/CT. The regions of interest (ROIs) were defined, and the mean standard uptake value (SUV_mean_) was recorded.

### Antitumor Effect In Vivo

After the tumor volume reached approximately 100 mm^3^, 4T1 tumor‐bearing mice were randomly divided into six groups (*n* = 5): 1) PBS, 2) X‐ray, 3) LND + X‐ray, 4) FPTM + X‐ray, 5) FPTM‐LP, 6) FPTM‐LP + X‐ray. Twelve hours after intravenously administration (100 µL, 10 mg kg^−1^), the corresponding groups was exposed to 6 Gy of X‐ray. Tumor dimension changes were measured and recorded within 16 d, and survival curves were drawn within 40 days. Additionally, tumors were harvested prior to fixation for pathological analysis (H&E, Ki67, caspase‐3, HIF‐1α, and TUNEL staining). Tumor volume = length×width×width/2

### Biological Safety Assessment

During the treatment period, the changes in body weight were recorded every 2 d. Blood samples were collected for serum biochemistry and hematological analyses. Major organs (lungs, liver, spleen, kidneys and heart) and tumors were excised for H&E staining.

### Statistical Analysis

Statistical analyses were conducted with GraphPad Prism 9.0.0. The CLSM images were analyzed using ImageJ 1.52a, and the PET/CT images were processed by PMOD 3.8. The quantitative data were expressed as mean ± standard deviation (s.d.) (*n* ≥ 3). Group comparisons were performed using unpaired two‐tailed student's *t* test, while multigroup comparisons were conducted using one‐way analysis of variance with Tukey's multiple comparisons. Survival curves were compared by the log‐rank test. (*p < 0.05, **p < 0.01, ***p < 0.001 and ****p < 0.0001).

## Conflict of Interest

The authors declare no conflict of interest.

## Supporting information



Supporting Information

## Data Availability

The data that support the findings of this study are available in the Supporting Information of this article.
