# Correction to “Nanoscale Metal‐Organic Framework‐Based Self‐Monitoring Oxygen Economizer and ROS Amplifier for Enhanced Radiotherapy‐Radiodynamic Therapy”

**DOI:** 10.1002/advs.76714

**Published:** 2026-07-17

S. Du, Q. Wen, T. Han, et al. “Nanoscale Metal‐Organic Framework‐Based Self‐Monitoring Oxygen Economizer and ROS Amplifier for Enhanced Radiotherapy‐Radiodynamic Therapy,” *Advanced Science* 12 no. 35 (2025): e03582, https://doi.org/10.1002/advs.202503582.

In the originally published article, we found that the Ki67 image of the tumor in the x‐ray group in Figure 8F was improperly used during the layout of the images. This correction does not affect the overall analysis, interpretation, or conclusions of the study. The corrected image is provided below.


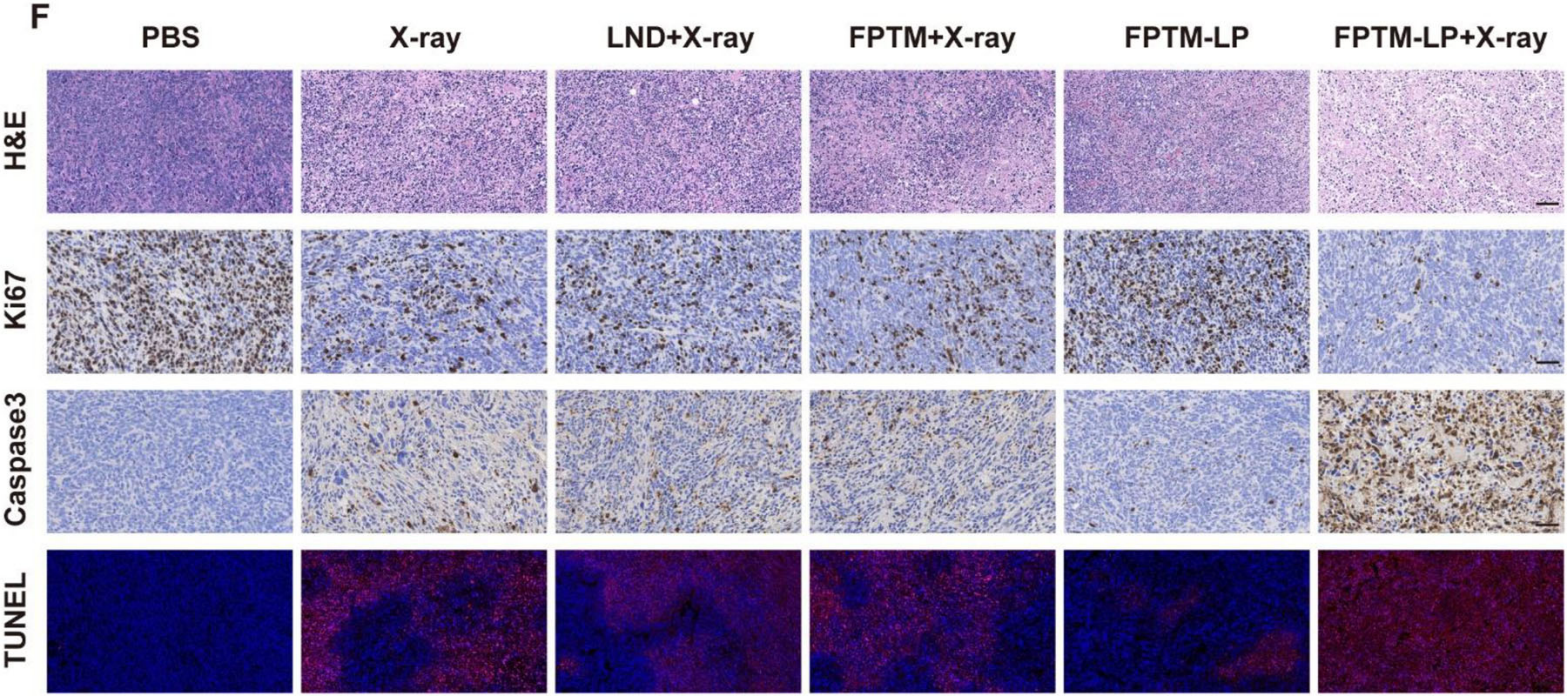


We apologize for this error.

