# Supplementary material for: Nanoscale Metal‐Organic Framework‐Based Self‐Monitoring Oxygen Economizer and ROS Amplifier for Enhanced Radiotherapy‐Radiodynamic Therapy
Source: Adv Sci (Weinh). 2025 Jun 23;12(35):e03582. doi: 10.1002/advs.202503582 (PMC12463018; doi:10.1002/advs.202503582)
Supplement: Supplementary file 1 — Supporting Information [file ADVS-12-e03582-s001.docx]

Supporting Information

Nanoscale Metal-Organic Framework-Based Self-Monitoring Oxygen Economizer and ROS Amplifier for Enhanced Radiotherapy-Radiodynamic Therapy

Shiye Du, Qiang Wen, Ting Han, Jiongyu Ren, Mingyu Wang, Yunpeng Dai, Xiaoguang Ge, Lu Li, Junzhi Liu*, and Shi Gao*


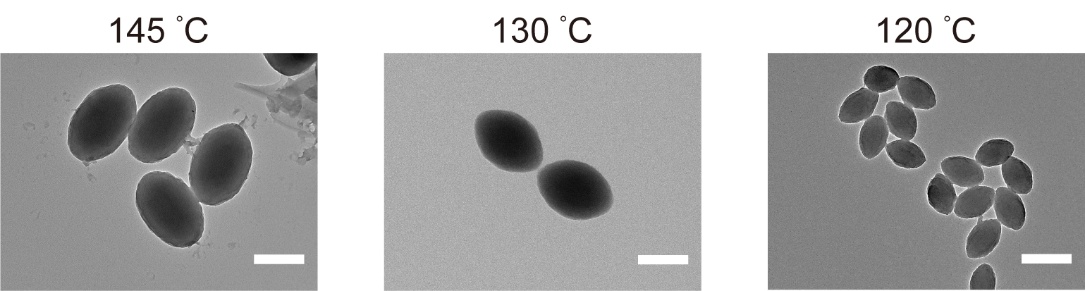


**Figure S1.** TEM image of FPTM at different reaction temperatures (Scale bar: 200 nm).


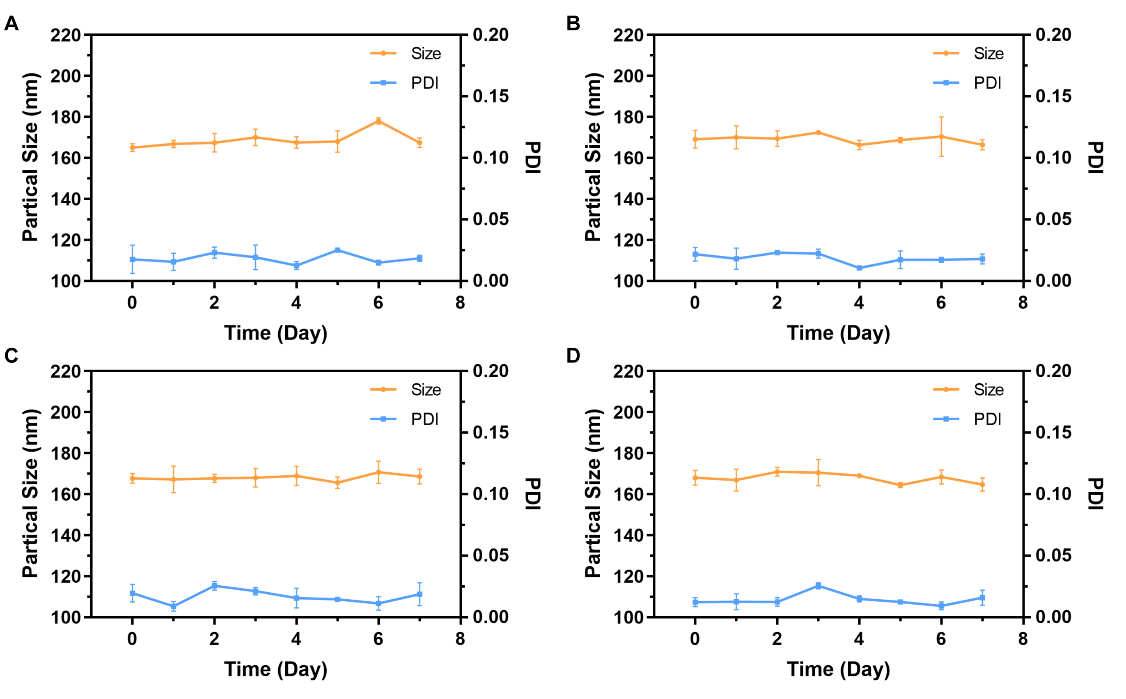


**Figure S2.** The stability of FPTM-LP in (A) PBS (pH 7.4), (B) serum-containing culture medium, (C) mouse serum and (D) distilled water. (n = 3, mean ± SD).


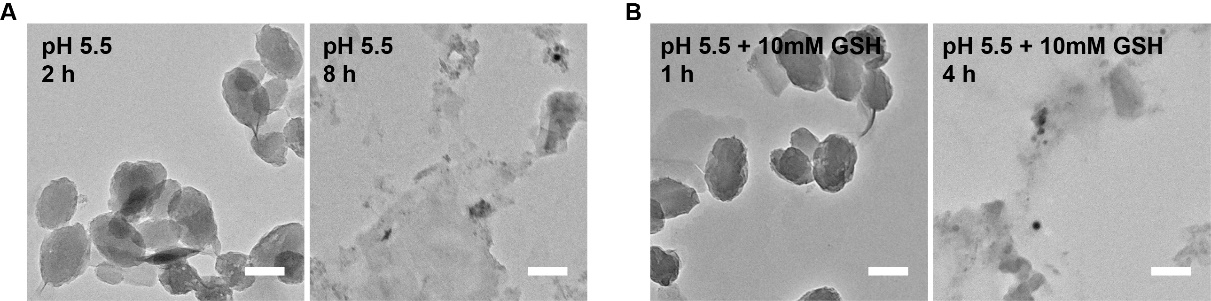


**Figure S3.** TEM images illustrating the effect of GSH/pH on the morphology of FPTM-LP at various exposure times (Scale bar: 100 nm).


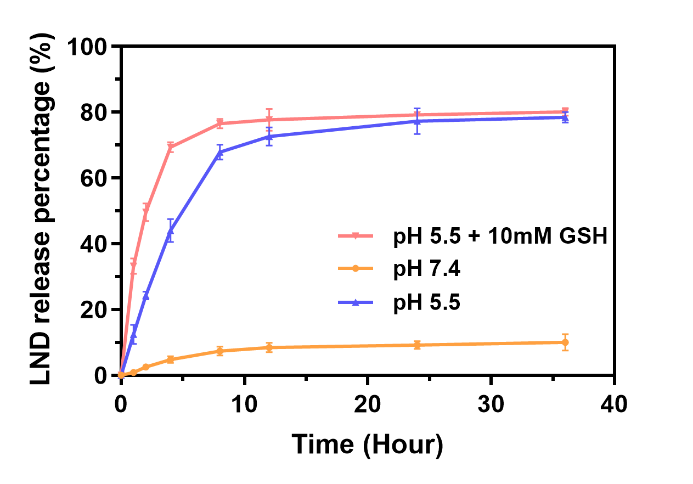


**Figure S4.** Release curves of LND under different conditions (n = 3, mean ± SD).


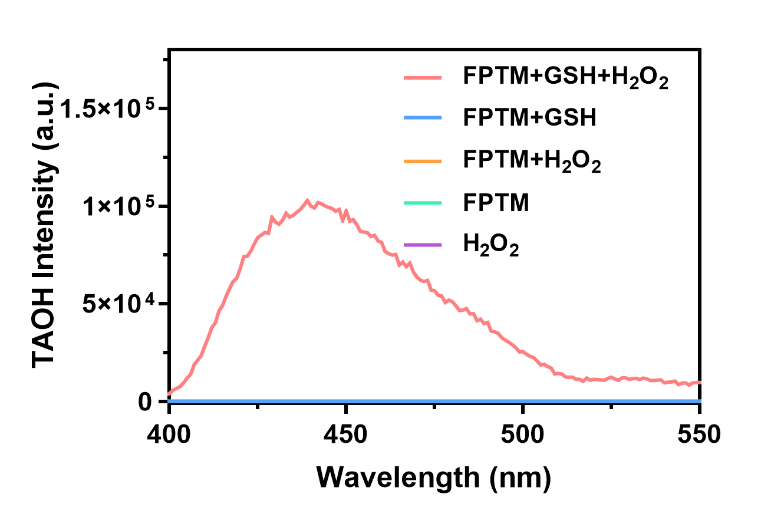


**Figure S5.** The fluorescence spectrum of TAOH indicates that FPTM can generate •OH through the Fenton reaction.


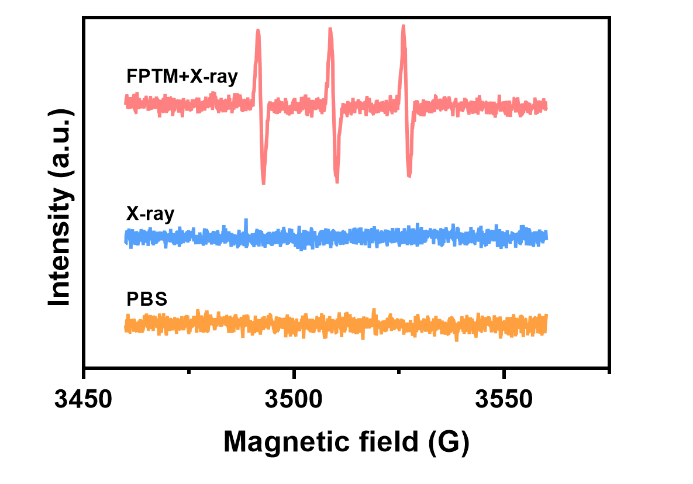


**Figure S6.** ESR spectra of FPTM to generate ^1^O_2_ under X-ray excitation.


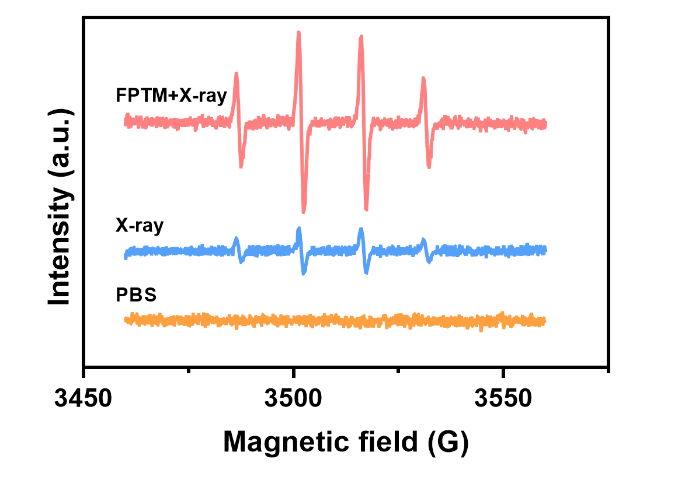


**Figure S7.** ESR spectra of FPTM to generate •OH under X-ray excitation.


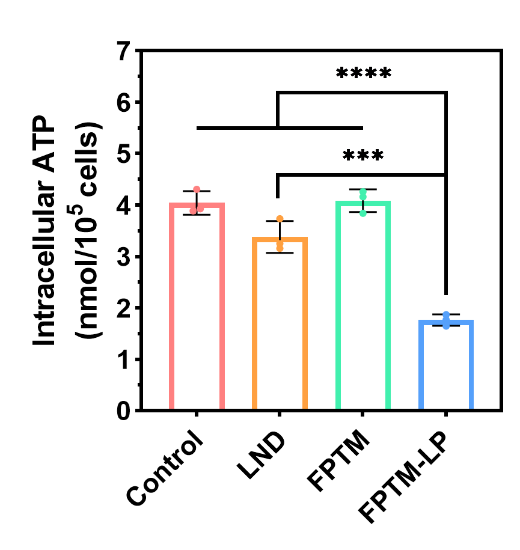


**Figure S8.** Intracellular ATP levels of 4T1 cells after different treatments (One-way ANOVA, n = 3, mean ± SD, ****p* < 0.001 and *****p* < 0.0001).


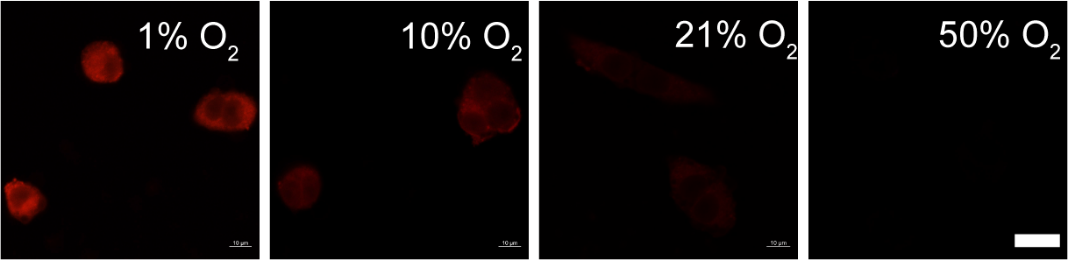


**Figure S9.** CLSM images of 4T1 cells after co-incubation with FPTM at different O_2_ concentrations (Scale bar: 20 μm).


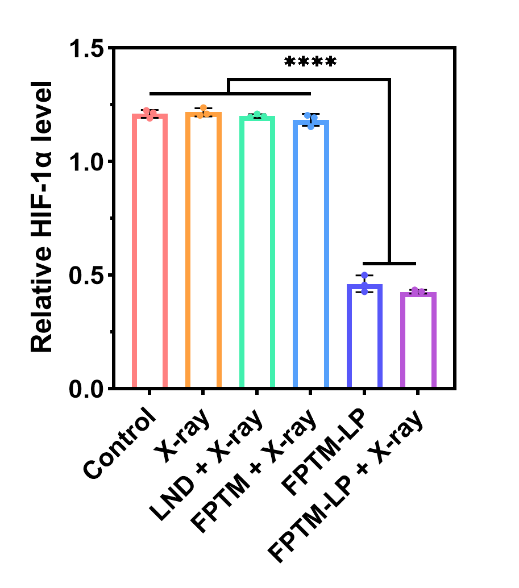


**Figure S10.** Quantitative analysis of HIF-1α Western blot results for each group after different treatments (One-way ANOVA, n = 3, mean ± SD, *****p* < 0.0001).


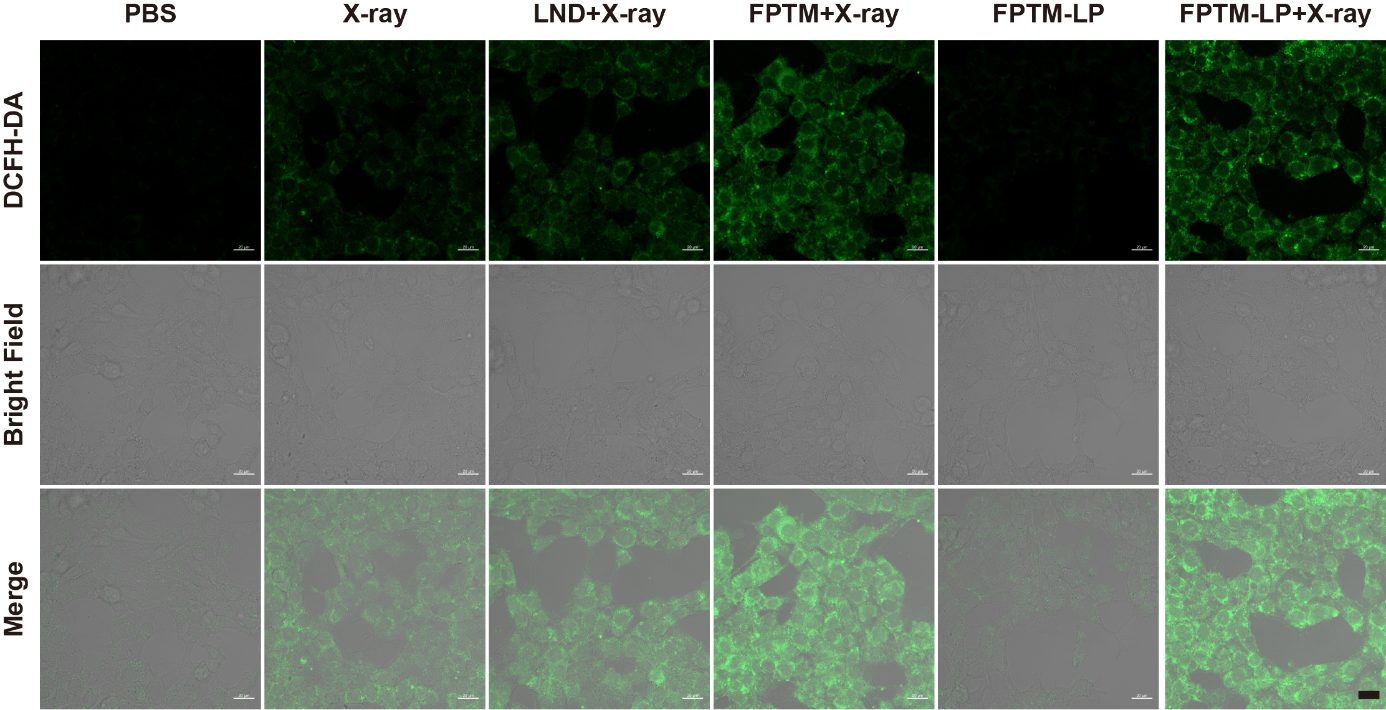


**Figure S11.** CLSM images of ROS levels detected by DCFH-DA under normoxic conditions (Scale bar: 20 μm).


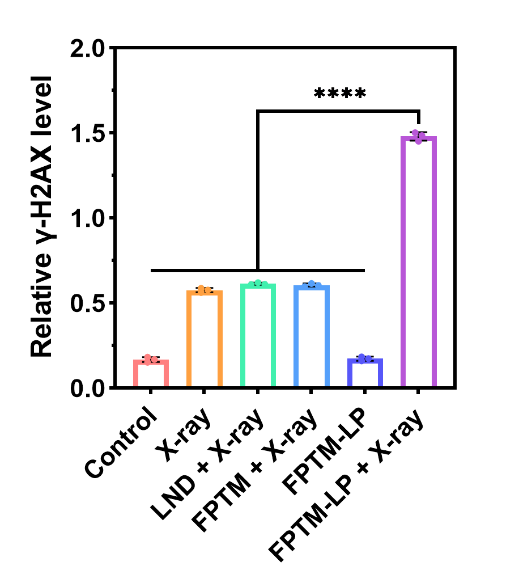


**Figure S12.** Quantitative analysis of γ-H2AX Western blot results for each group after different treatments (One-way ANOVA, n = 3, mean ± SD, *****p* < 0.0001).


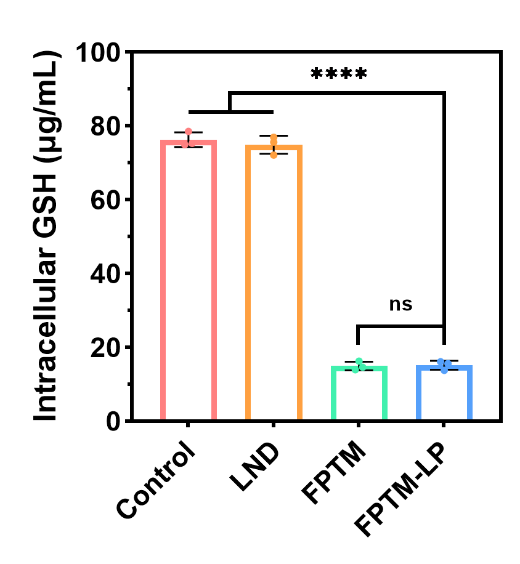


**Figure S13.** Intracellular GSH levels of 4T1 cells after different treatments (One-way ANOVA, n = 3, mean ± SD, *****p* < 0.0001).


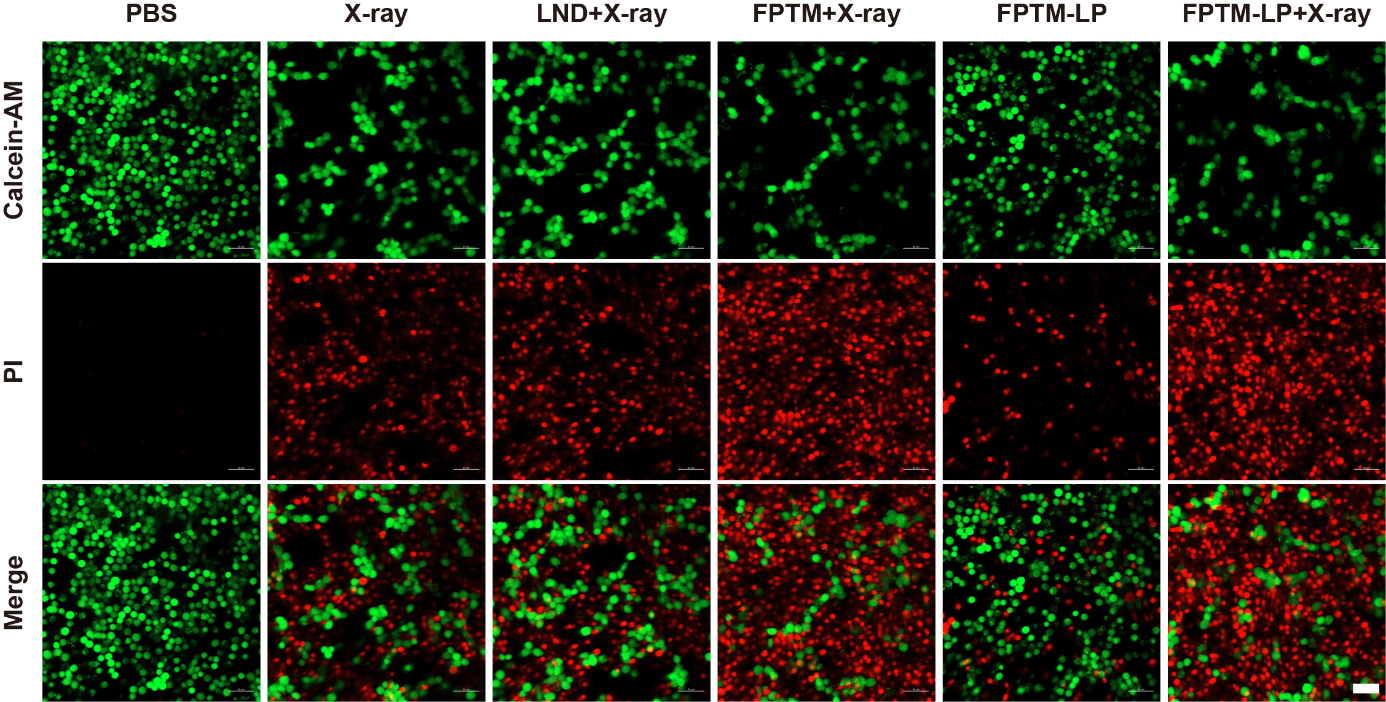


**Figure S14.** CLSM images of calcein-AM/PI staining under normoxic conditions (Scale bar: 50 μm).


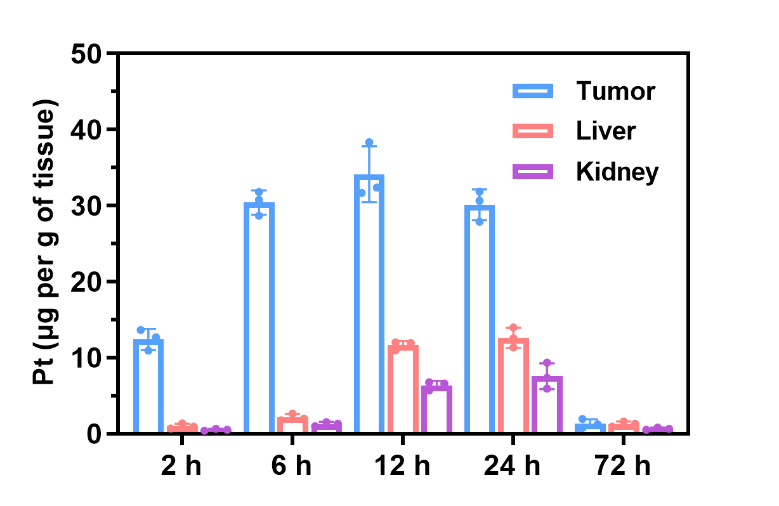


**Figure S15.** Variations in Pt(II) content at different time points in tumor, liver, and kidneys (n = 3, mean ± SD).


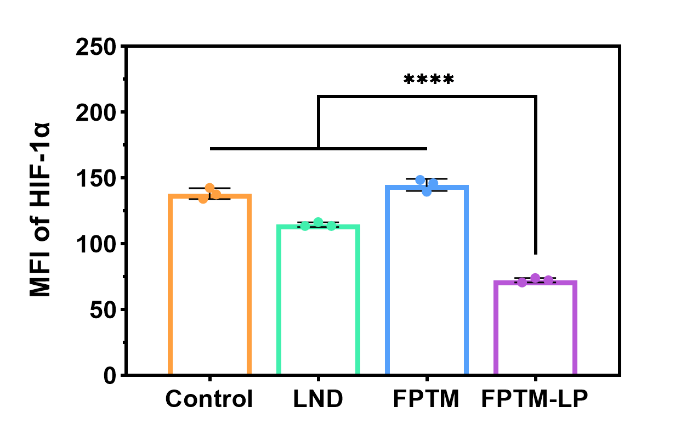


**Figure S16.** Average fluorescence intensity of HIF-1α in tumor sections of each group after different treatments (One-way ANOVA, n = 3, mean ± SD, *****p* < 0.0001).


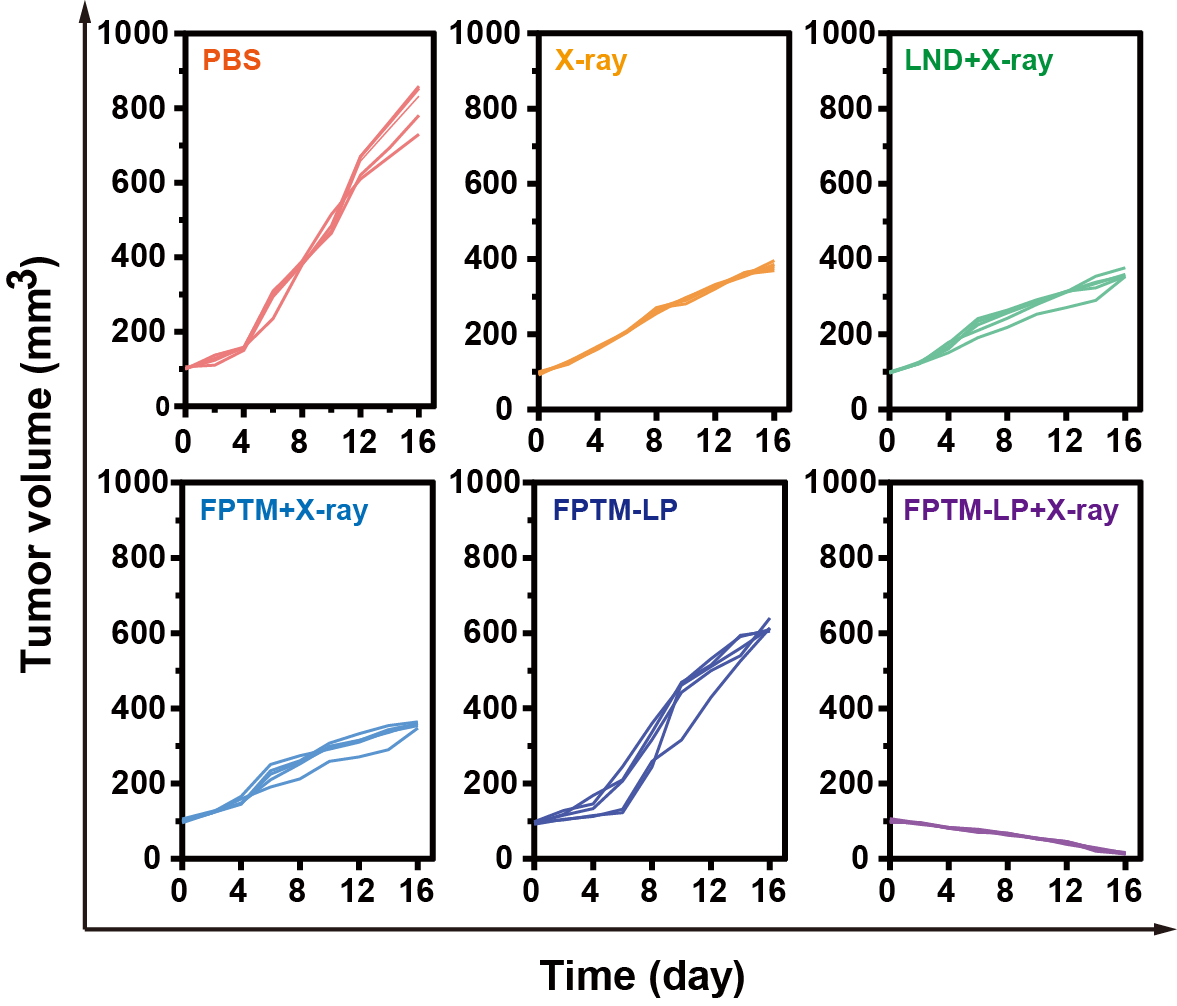


**Figure S17.** The curves of tumor volume changes in mice from various groups during the treatment process (n = 5).


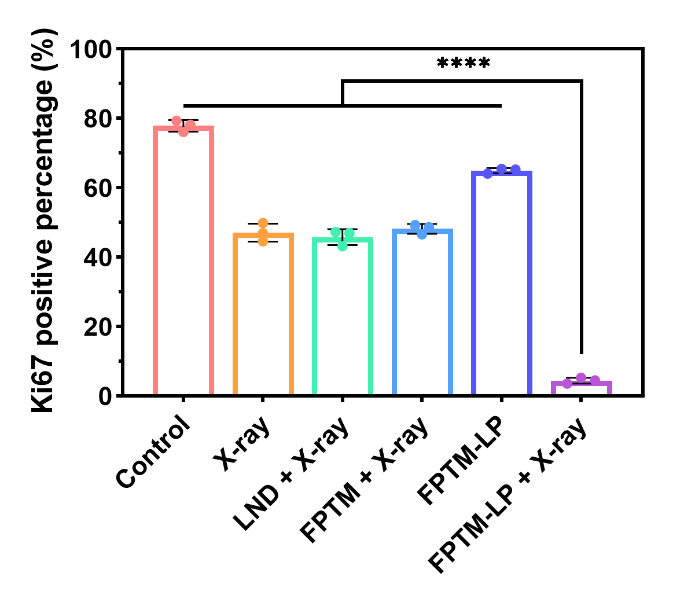


**Figure S18.** Semi-quantitative analysis of the Ki67-positive cell rate in tumor sections of each group after different treatments (One-way ANOVA, n = 3, mean ± SD, *****p* < 0.0001).


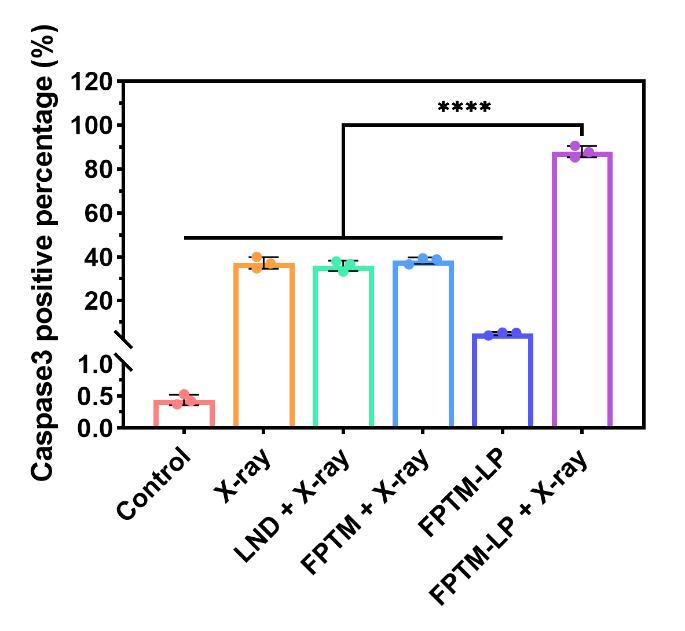


**Figure S19.** Semi-quantitative analysis of the Caspase3-positive cell rate in tumor sections of each group after different treatments (One-way ANOVA, n = 3, mean ± SD, *****p* < 0.0001).


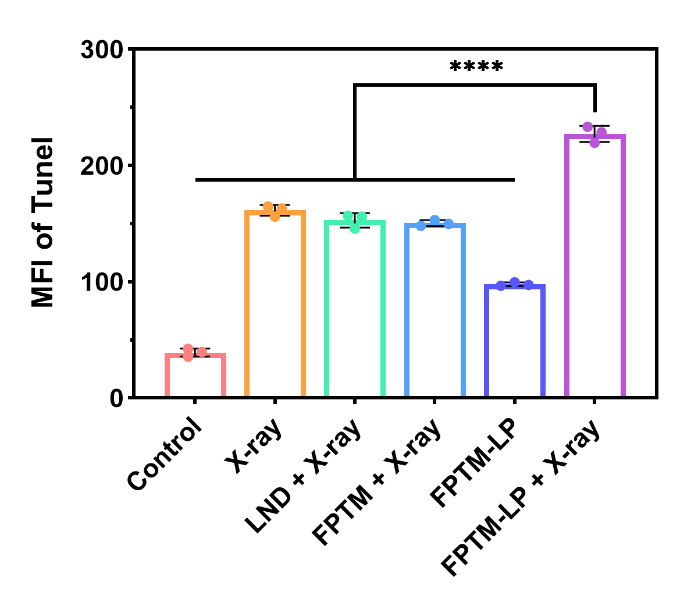


**Figure S20.** Average fluorescence intensity of TUNEL in tumor sections of each group after different treatments (One-way ANOVA, n = 3, mean ± SD, *****p* < 0.0001).


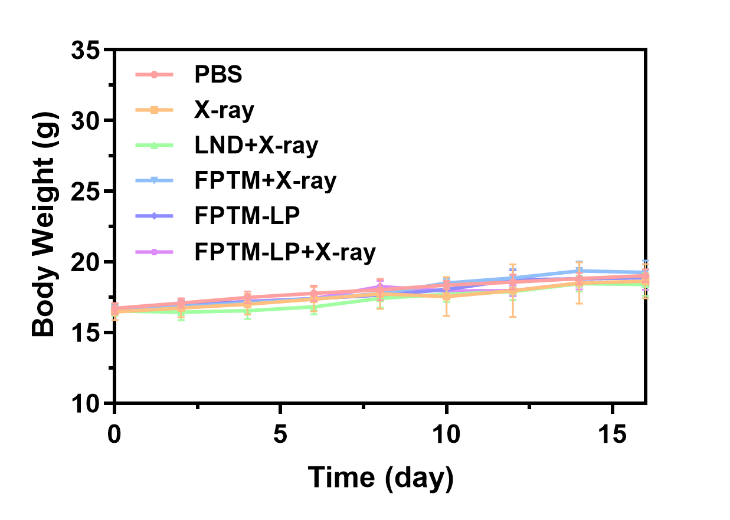


**Figure S21.** Weight changes of mice in various groups during the treatment process (n = 5, mean ± SD).

**Table S1.** Tumor weights of each group at the end of treatment.

| Sample | PBS  [g] | X-ray  [g] | LND  + X-ray [g] | FPTM  + X-ray  [g] | FPTM-LP [g] | FPTM-LP  + X-ray [g] |
| --- | --- | --- | --- | --- | --- | --- |
| 1 | 0.69 | 0.39 | 0.32 | 0.34 | 0.41 | 0.05 |
| 2 | 0.62 | 0.38 | 0.32 | 0.32 | 0.42 | 0.03 |
| 3 | 0.58 | 0.39 | 0.33 | 0.34 | 0.39 | 0.03 |
| 4 | 0.59 | 0.37 | 0.34 | 0.32 | 0.40 | 0.04 |
| 5 | 0.57 | 0.35 | 0.31 | 0.31 | 0.41 | 0.03 |

**Table S2.** The changes in survival rates over time for each group.

| Time  [d] | PBS  [%] | X-ray  [%] | LND  + X-ray  [%] | FPTM  + X-ray  [%] | FPTM-LP  [%] | FPTM-LP  + X-ray  [%] |
| --- | --- | --- | --- | --- | --- | --- |
| 19 | 80 | 100 | 100 | 100 | 100 | 100 |
| 20 | 60 | 100 | 100 | 100 | 100 | 100 |
| 21 | 20 | 100 | 100 | 100 | 60 | 100 |
| 22 | 20 | 100 | 80 | 100 | 60 | 100 |
| 23 | 20 | 100 | 80 | 100 | 40 | 100 |
| 25 | 0 | 100 | 80 | 100 | 20 | 100 |
| 26 | 0 | 100 | 80 | 100 | 0 | 100 |
| 27 | 0 | 100 | 60 | 100 | 0 | 100 |
| 28 | 0 | 100 | 40 | 80 | 0 | 100 |
| 30 | 0 | 60 | 40 | 40 | 0 | 100 |
| 34 | 0 | 40 | 40 | 40 | 0 | 100 |
| 36 | 0 | 20 | 40 | 40 | 0 | 100 |
| 40 | 0 | 20 | 40 | 40 | 0 | 100 |

**Table S3.** Blood biochemistry and blood routine analysis after treatment.

| Hematological/  serum biochemical indicators | PBS | FPTM-LP | FPTM-LP  + X-ray | Reference |
| --- | --- | --- | --- | --- |
| ALT [U/L] | 112 ± 14.53 | 115.67 ± 16.2 | 122 ± 2.65 | 28-132 |
| AST [U/L] | 202.33 ± 8.15 | 219 ± 24.3 | 217.33 ± 29.02 | 59-247 |
| CK [U/L] | 566 ± 18.08 | 567.67 ± 38.7 | 566 ± 16.09 | 68-1070 |
| LDH [U/L] | 552 ± 8.54 | 544 ± 34.78 | 575.67 ± 15.89 | 100-1000 |
| CREA [μmol/L] | 32 ± 4.58 | 33 ± 1 | 31.33 ± 4.04 | 18-71 |
| UREA [mmol/L] | 8.5 ± 0.27 | 9.4 ± 0.27 | 9.1 ± 0.5 | 6.4-10.4 |
| WBC [10^9^/L] | 8.74 ± 0.25 | 8.66 ± 0.11 | 8.49 ± 0.38 | 0.8-10.6 |
| Gran [%] | 36.17 ± 2.56 | 34.1 ± 2.43 | 34.3 ± 0.96 | 6.5-50 |
| Lymph [%] | 45.97 ± 2.58 | 47.9 ± 2.04 | 48.1 ± 1.21 | 40-92 |
| Mon [%] | 10.9 ± 0.2 | 11.27 ± 0.15 | 11.1 ± 0.3 | 0.9-18 |
| Eo [%] | 5.77 ± 0.35 | 5.67 ± 0.32 | 5.4 ± 0.3 | 0-7.5 |
| Baso [%] | 1.2 ± 0.1 | 1.07 ± 0.15 | 1.1 ± 0.2 | 0-1.5 |
| RBC [10^12^/L] | 9.4 ± 0.49 | 9.62 ± 0.32 | 9.26 ± 0.45 | 6.5-11.5 |
| HGB [g/L] | 150 ± 5 | 153.33 ± 7.37 | 154 ± 9.64 | 110-165 |
| HCT [%] | 42.07 ± 0.81 | 42.6 ± 0.78 | 42.77 ± 0.81 | 35-55 |
| MCV [fL] | 45.4 ± 0.92 | 45.6 ± 1.25 | 45.9 ± 1.15 | 41-55 |
| MCH [pg] | 16.27 ± 0.15 | 16.13 ± 0.38 | 16.63 ± 0.21 | 13-18 |
| MCHC [g/L] | 352.67 ± 3.01 | 349.67 ± 4.04 | 350 ± 2 | 300-360 |
| RDW-CV [%] | 16.93 ± 0.72 | 17.17 ± 0.78 | 17.17 ± 0.12 | 12-19 |
| RDW-SD [fL] | 35.37 ± 2.01 | 34.67 ± 0.7 | 35.57 ± 0.61 | 23-39 |
| PLT [10^9^/L] | 644 ± 12.49 | 654 ± 18.19 | 675 ± 5.57 | 400-1600 |
| MPV [fL] | 5.9 ± 0.35 | 5.8 ± 0.3 | 5.77 ± 0.31 | 4-6.2 |
| PDW [fL] | 15.83 ± 0.12 | 15.73 ± 0.15 | 15.67 ± 0.32 | 12-17.5 |
| PCT [%] | 0.38 ± 0.03 | 0.36 ± 0.02 | 0.34 ± 0.04 | 0.1-0.78 |
